# The Regenerative Effect of Trans-spinal Magnetic Stimulation After Spinal Cord Injury: Mechanisms and Pathways Underlying the Effect

**DOI:** 10.1007/s13311-020-00915-5

**Published:** 2020-08-27

**Authors:** C. Chalfouh, C. Guillou, J. Hardouin, Q. Delarue, X. Li, C. Duclos, D. Schapman, J.-P. Marie, P. Cosette, N. Guérout

**Affiliations:** 1 Normandie Univ, UNIROUEN, EA3830 GRHV, 76000 Rouen, France; 2 Institute for Research and Innovation in Biomedicine (IRIB), 76000 Rouen, France; 3 PISSARO Proteomic Facility, Normandie Univ, UNIROUEN, 76821 Mont-Saint-Aignan, France; 4 Institute for Research and Innovation in Biomedicine (IRIB), Mont-Saint-Aignan, France; 5 Department of Neurobiology, Care sciences and Society, BioClinicum, Karolinska Institutet, 17164 Stockholm, Sweden; 6 Normandie Univ, UNIROUEN, SFR IRIB, Plateau PRIMACEN, F-76821 Mont-Saint-Aignan, France

**Keywords:** Rehabilitation, spinal cord injury, glial scar, magnetic stimulation and neuroregeneration

## Abstract

Spinal cord injury (SCI) leads to a loss of sensitive and motor functions. Currently, there is no therapeutic intervention offering a complete recovery. Here, we report that repetitive trans-spinal magnetic stimulation (rTSMS) can be a noninvasive SCI treatment that enhances tissue repair and functional recovery. Several techniques including immunohistochemical, behavioral, cells cultures, and proteomics have been performed. Moreover, different lesion paradigms, such as acute and chronic phase following SCI in wild-type and transgenic animals at different ages (juvenile, adult, and aged), have been used. We demonstrate that rTSMS modulates the lesion scar by decreasing fibrosis and inflammation and increases proliferation of spinal cord stem cells. Our results demonstrate also that rTSMS decreases demyelination, which contributes to axonal regrowth, neuronal survival, and locomotor recovery after SCI. This research provides evidence that rTSMS induces therapeutic effects in a preclinical rodent model and suggests possible translation to clinical application in humans.

**Electronic supplementary material:**

The online version of this article (10.1007/s13311-020-00915-5) contains supplementary material, which is available to authorized users.

## Background

Spinal cord injury (SCI) is an incurable disease which leads to a permanent loss of motor, sensation, and sensory functions below the injury level [1]. To date, no cure can be offered to paraplegic or tetraplegic patients. Thus, for many decades, researchers have conducted a substantial number of studies in order to understand the mechanisms responsible for the lack of recovery following SCI. Thereby, it has been reported that SCI induces multiple cellular and molecular responses as inflammation, neuronal death, demyelination, and scar formation [2], which lead to limited axonal regrowth and recovery. However, the distinct role of each cellular population in the spinal cord has been recently investigated and described after SCI [3]. Specifically, the scar which occurs after SCI [4], mainly described as an inhibitory glial scar, is formed by not only astrocytes but also fibroblasts with cell type–specific contributions [5]. Indeed, the astrocytic scar exerts mainly protective effects by restricting the spread of inflammation and secreting permissive molecules constituting a bridge for axonal regrowth [6]. On the contrary, the fibrotic scar exerts inhibitory effects on axonal regrowth and functional recovery [7]. More recently, by using optogenetic tools, it has been demonstrated that the inhibition of the fibrotic scar formation promotes axonal regrowth and functional recovery after SCI [8]. Furthermore, it has been shown that the administration of specific growth factors, such as, osteopontin, insulin-like growth factor 1, and ciliary-derived neurotrophic factor, can induce axonal regrowth across the lesion core [9]. In addition, the endogenous neural stem cells in the spinal cord can also contribute to the scar formation, and secrete neurotrophic factors to prevent neuronal death [10]. These quiescent adult neural stem cells in the spinal cord can be activated after SCI, and differentiate into mostly astrocytes and migrate to the injury site to form the core of astrocytic scar [10, 11]. Therefore, ependymal cells have been proposed as a therapeutic target for endogenous regeneration after SCI [11, 12]. These recent discoveries have led to new starting points for innovative therapies based on the modulation of the cellular populations in the lesion scar. However, as most of the studies use cellular transplantations or genetic modulations of lesion scar for axonal regeneration and functional recovery, their administration protocols are complex for clinical context due to their invasiveness, endogenous genetic manipulation, potential side effects, and/or pre-injury characteristics [6, 7, 13]. Therefore, a noninvasive treatment combining the modulation of endogenous cell types is of high interest for SCI therapy.

Repetitive transcranial magnetic stimulation (rTMS) has been already used clinically to treat neuropsychiatric diseases [14] and is considered a promising future treatment for stroke [15], Parkinson’s [16], or Alzheimer’s diseases [17], despite that the mechanisms behind is not fully understood. It has been hypothesized that magnetic fields exert neuroprotective [18, 19] and remodeling effects on the tissues [20]. Although new in vitro and in vivo studies have demonstrated that rTMS induces changes of gene expression related to apoptosis and neurite outgrowth [21, 22], very few studies have explored the benefit of the rTMS following SCI. In particular, none has so far used a focal and noninvasive repetitive trans-spinal magnetic stimulation (rTSMS) paradigm as treatment after SCI, neither are the cellular and molecular mechanisms elucidated.

In this study, we propose that a noninvasive treatment, the focal rTSMS, has neuroprotective and neuroregenerative effects on the injured spinal cord. By using wild-type (WT) and transgenic animals at different ages, in vivo and in vitro methods, and proteomics, we evaluated the recovery effects of this therapy, and investigated the modulation of the scar, and the cellular and molecular mechanisms. Our study suggests that rTSMS can modulate the scar formation and support neuronal survival as well as promote regeneration. Therefore, we propose it as a potential noninvasive SCI therapy, for patients at different ages.

## Methods

### Experimental Design

**Fig. 1:**
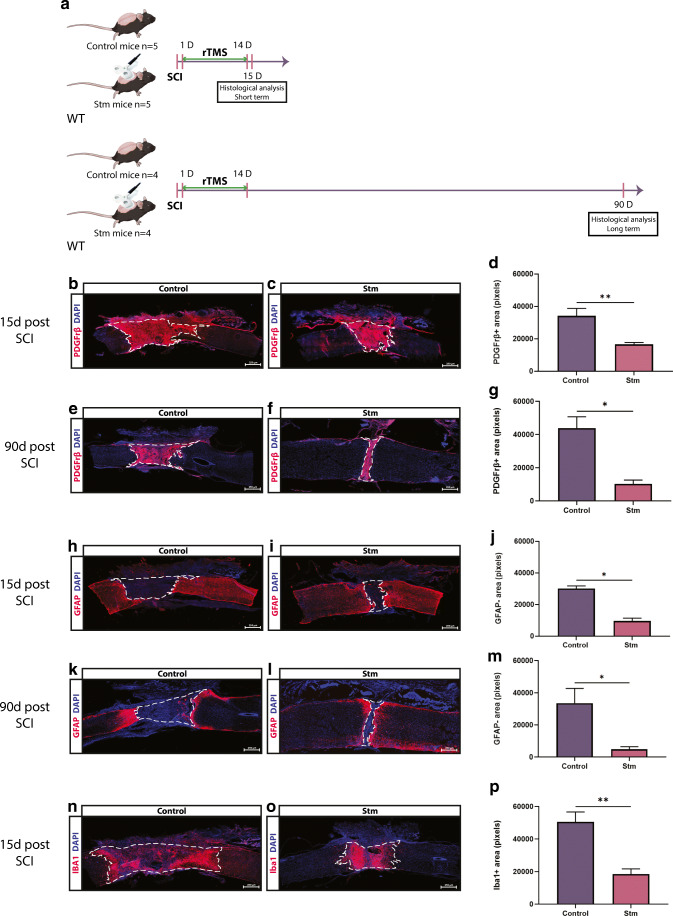
rTSMS-based treatment modulates spinal scar formation after SCI. (A) Experimental design, SCIs were performed on mice (day 0), then half of the mice has been treated using rTSMS during 2 weeks (from day 1 to day 14). At day 15 and day 90, mice were fixed and immunohistological analyses were performed. At day 15 (B–D, H–J, N–P) and day 90 (E–G, K–M), two groups of mice were fixed and immunohistological analyses were performed. (B, C, E, F, H, I, K, L, N, O) Representative pictures of sagittal spinal cord sections of (B, E, H, K, N) control (SCI) and (C, F, I, L, O) Stm (rTSMS treated) animals 15 and 90 days after SCI. Sections were stained with (B, C, E, F) PDGFRβ, (H, I, K, L) GFAP, (N, O) Iba1, and DAPI. (B–D, E–G) Fibrosis-positive area (PDGFRβ+) and quantification of PDGFRβ+ area 15 and 90 days after SCI. (H–J, K–M) Astrocytic-negative area (GFAP−) and quantification of GFAP− area 15 and 90 days after SCI. (N–P) Iba1-positive area (Iba1+) and quantification of Iba1+ area 15 days after SCI. Scale bars are 200 μm. *N* = 5 animals per group at 15 days and *N* = 4 animals per group at 90 days. Quantifications are expressed as average ± SEM. * = *p* < 0.05; ** = *p* < 0.01

**Fig. 2:**
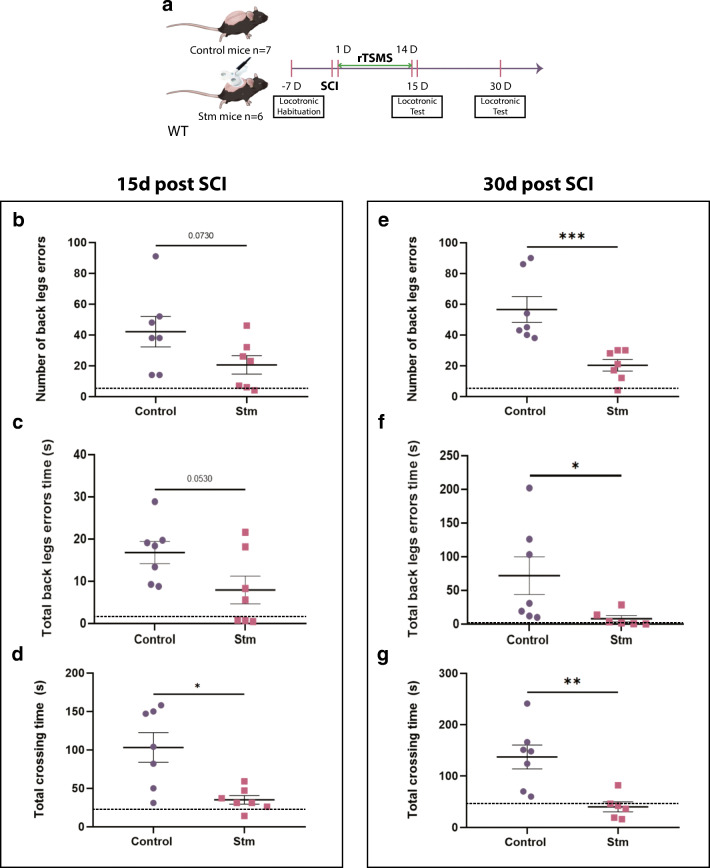
rTSMS-based treatment enhances functional recovery. (A) Experimental design, SCIs were performed on mice (day 0), then half of the mice has been treated using rTSMS during 2 weeks (from day 1 to day 14). At day 15 and day 30, functional recovery was analyzed using locotronic test. (B–G) At days 15 and 30, functional recovery was analyzed for the two groups of mice using locotronic test. (B, E) Quantification of the number of back leg errors 15 and 30 days after SCI. (C, F) Quantification of the total back leg error time 15 and 30 days after SCI. (D, G) Quantification of the total crossing time 15 and 30 days after SCI. *N* = 6–7 animals per group. Dashed lines correspond to the baseline parameters obtained during locotronic habituation (7 days before SCI). Quantifications are expressed as average ± SEM. * = *p* < 0.05; ** = *p* < 0.01, *** = *p* < 0.001

An overview of the paradigms used in this study is presented in Figs. 1A, 2A, 5A, 6A, 7A, and 8A.

**Fig. 3:**
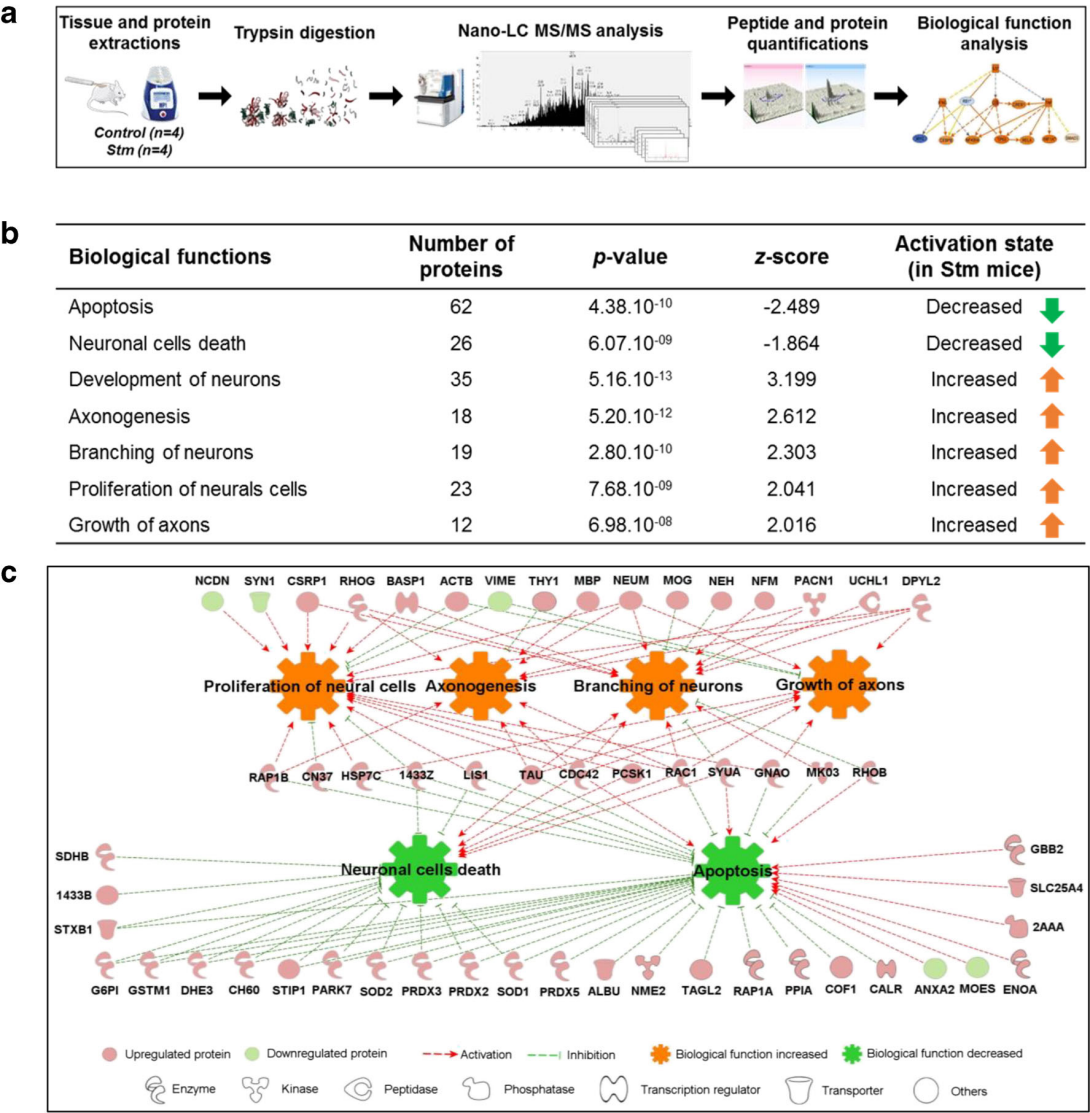
rTSMS regulates functional protein synthesis and pathways. (A) Workflow for quantitative proteomics and data analyses of spinal cord tissue between stimulated (rTSMS treated) and control (SCI) mice. (B) Modulation of biological functions involved in neural development and cell death from differentially regulated proteins in stimulated mice compared to controls. Analysis was performed using Ingenuity Pathway Analysis (IPA, Qiagen). Statistical significance is proposed through the calculation of *p* values and *z*-scores. (C) Functional regulatory effects of upregulated (red) or downregulated (green) proteins belonging to the different pathways associated to neural development and cell death (red arrows: activation; green arrows: inhibition; orange nut: gain of biological function; green nut: decrease of biological function)

**Fig. 4:**
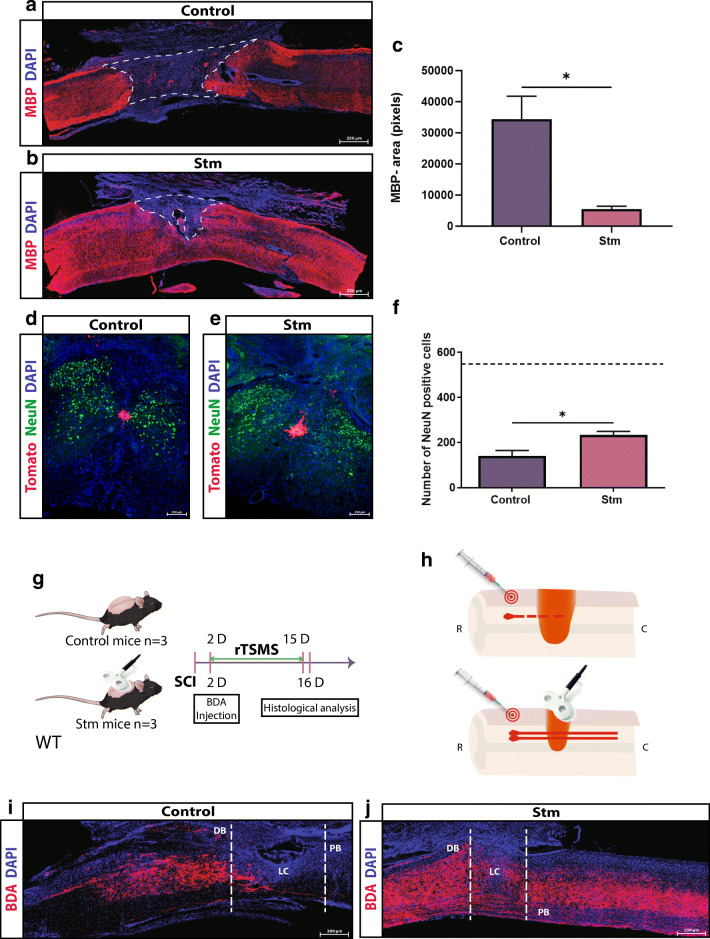
rTSMS inhibits demyelination and enhances neuronal survival and axonal regrowth. (A, B) Representative pictures of sagittal spinal cord sections of (A) control (SCI) and (B) Stm (rTSMS treated) animals 15 days after SCI. Sections were stained with MBP and DAPI. (C) Quantification of MBP-negative area (MBP−) 15 days after SCI. Scale bars are 200 μm. *N* = 5–6 animals per group. (D, E) Representative pictures of axial spinal cord sections of (D) control and (E) Stm animals 15 days after SCI. Sections were stained with NeuN and DAPI. (F) Quantification of NeuN+ cells 15 days after SCI. Scale bars are 200 μm. Dashed line corresponds to the number of NeuN+ cells from non-injured spinal cord. *N* = 4 animals per group. (G, H) Design of the BDA-labeled axons experiments. SCIs were performed on mice (day 0), 2 days after mice have been injected with BDA. Then, half of the mice has been treated using rTSMS during 2 weeks (from day 2 to day 15). At day 16, mice were fixed and immunohistological analyses were performed on control and Stm mice. (I, J) Representative pictures of sagittal spinal cord sections of (I) control and (J) Stm mice 16 days after SCI. Sections were stained with BDA and DAPI. Dashed lines demarcate proximal (PB) and distal (DB) borders around lesion core (LC). Images are representative of *n* = 3 animals per group. Scale bars are 200 μm. Quantifications are expressed as average ± SEM. * = *p* < 0.05

**Fig. 5:**
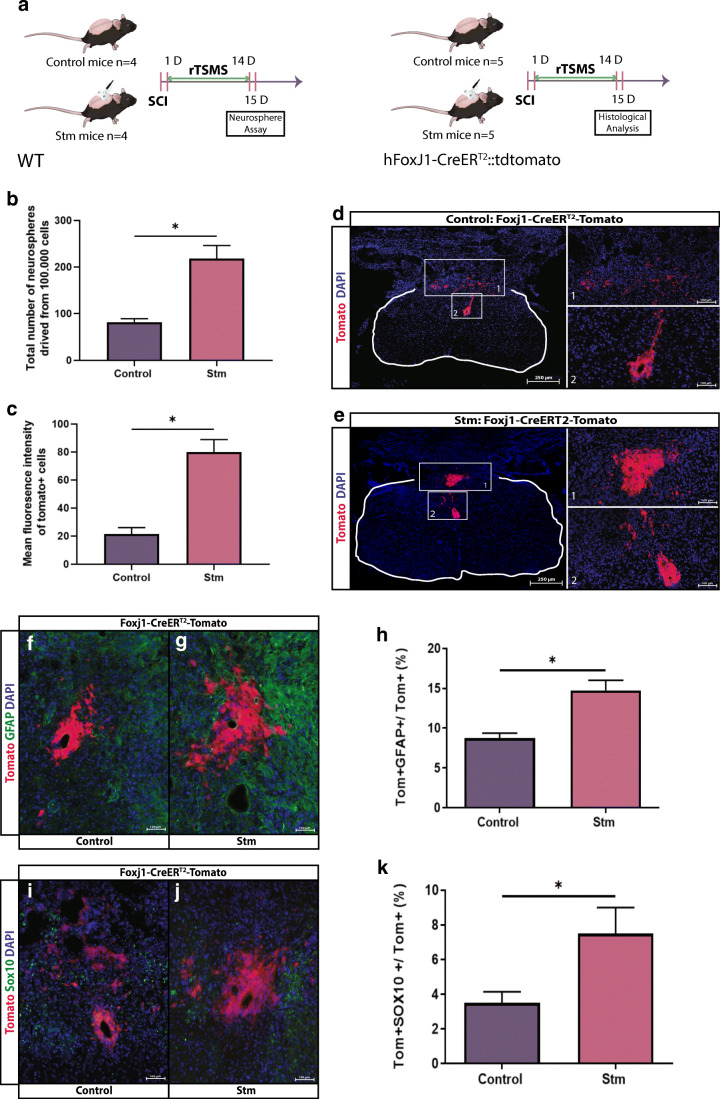
rTSMS increases proliferation and modulates differentiation of spinal cord stem cells. (A) Experimental design, SCIs were performed on mice (day 0), then half of the mice has been treated using rTSMS during 2 weeks (from day 1 to day 14). At day 15, mice were fixed and neurosphere assays (B) or immunohistological analyses (C–K) were performed. (B) Quantification of the total number of neurospheres derived from 100,000 cells from control (SCI) and Stm (rTSMS treated) mice 15 days after injury. *N* = 4 animals per group. (C) Quantification of mean fluorescence intensity of tomato+ cells 15 days after SCI. (D, E, F, G, I, J) Representative pictures of axial spinal cord sections of (D, F, I) control and (E, G, J) Stm animals 15 days after SCI. Sections were stained with tomato and DAPI (D, E). Scale bars are 250 μm. (F–K) Identification of cells derived from Tomato+ recombined ependymal cells were identified by GFAP (F, G) and Sox10 (I, J). (H, K) Quantification of double positive recombined cells in the spinal cord 15 days after SCI. Scale bars are 100 μm. *N* = 5 animals per group. Quantifications are expressed as average ± SEM. * = *p* < 0.05

Briefly, in Figs. 1, 2, 3, 4, and 5, SCIs were performed on mice (day 0), then half of the mice has been treated using rTSMS during 2 weeks (from day 1 to day 14).

In Figs. 1, 4, and 5, at day 15, mice were fixed and immunohistological analyses were performed. In addition, in Fig. 1 at day 90, two other groups of mice were fixed and immunohistological analyses were performed.

In Fig. 2, at day 15 and day 30, functional recoveries were analyzed using locotronic test.

In Fig. 3, at day 15, mice were sacrificed, and proteomic analyses were performed.

**Fig. 6:**
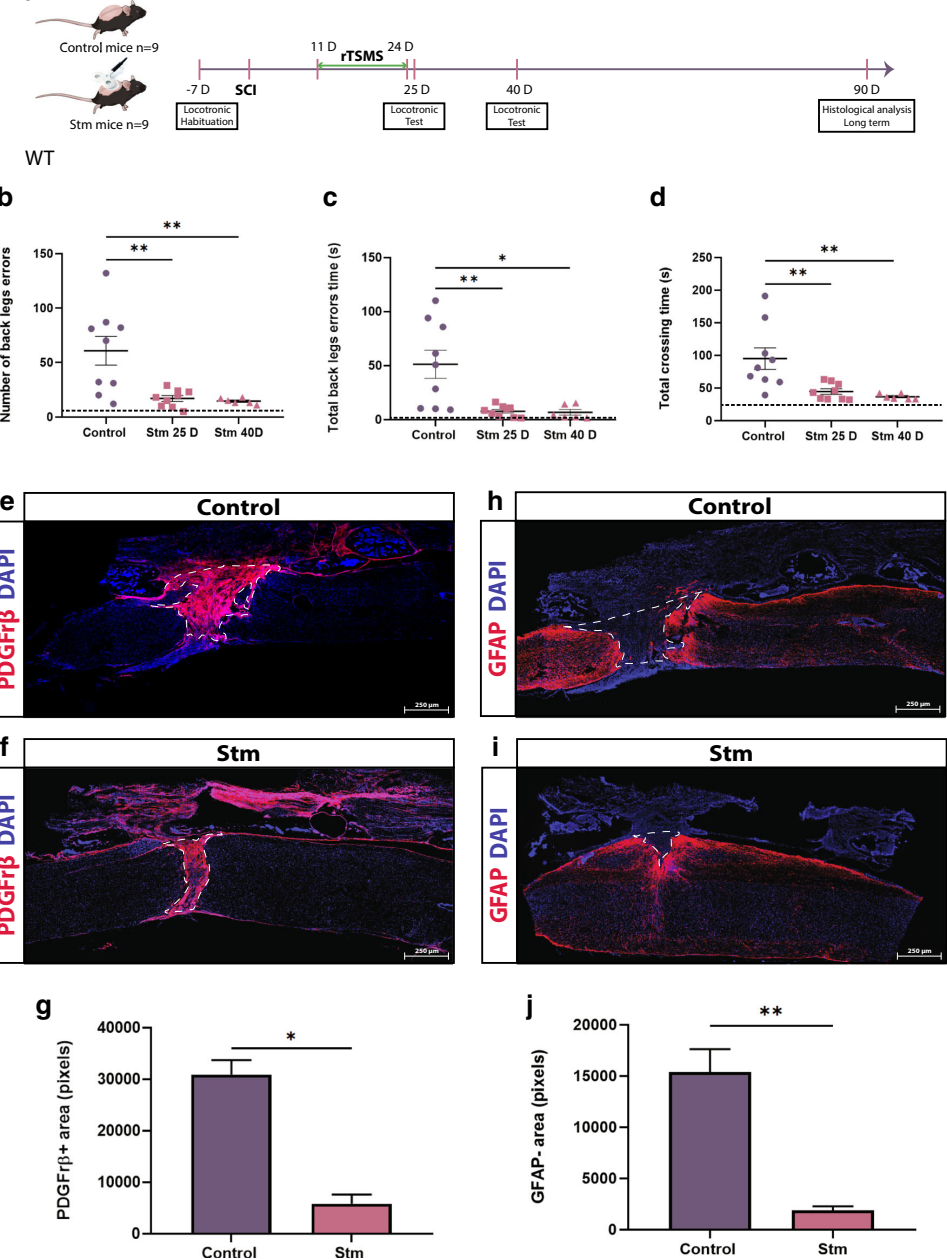
rTSMS-based treatment enhances functional recovery in a chronic condition. (A) Experimental design, SCIs were performed on mice (day 0), then 11 days after the same mice have been treated using rTSMS during 2 weeks (from day 11 to day 24). At days 25 and 40, functional recovery was analyzed using locotronic test. At day 90, mice were fixed and immunohistological analyses were performed. (B) Quantification of the number of back leg errors 25 days and 40 days after SCI. (C) Quantification of the total back leg error time 25 days and 40 days after SCI. (D) Quantification of the total crossing time 25 and 40 after SCI. *N* = 9 animals at 25 days and *N* = 6 animals at 40 days. Dashed lines correspond to the baseline parameters obtained during locotronic habituation (7 days before SCI). (E–J) At day 90, mice were fixed and immunohistological analyses were performed and Stm (rTSMS treated) animals were compared to control (SCI) mice. (E, F, H, I) Representative pictures of sagittal spinal cord sections of (E, H) control and (F and I) Stm mice 90 days after SCI. Sections were stained with (E, F) PDGFRβ and (H, I) GFAP and DAPI. (E–G) Fibrosis-positive area (PDGFRβ+) and quantification of PDGFRβ+ area 90 days after SCI. (H–J) Astrocytic-negative area (GFAP−) and quantification of GFAP− area 90 days after SCI. Scale bars are 200 μm. *N* = 4 animals per group. Quantifications are expressed as average ± SEM. * = *p* < 0.05; ** = *p* < 0.01

**Fig. 7:**
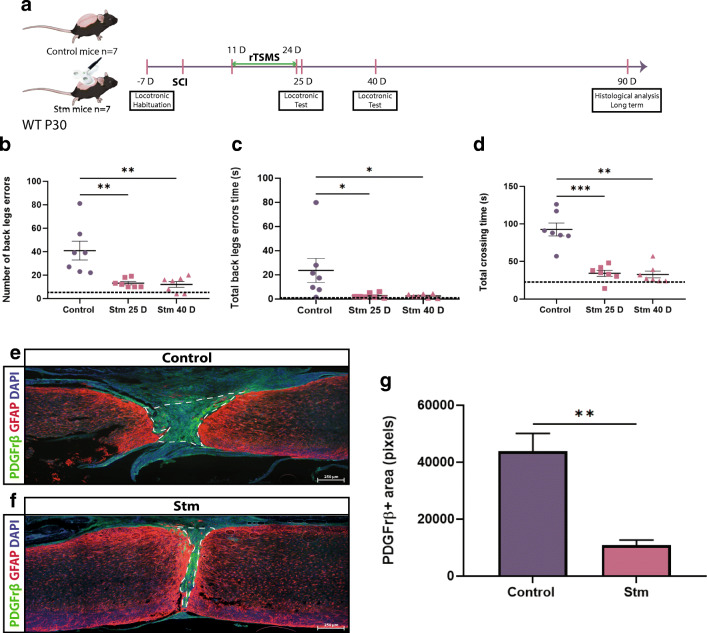
rTSMS-based treatment enhances functional recovery and modulates scar formation in a chronic condition in juvenile mice. (A) Experimental design, SCIs were performed on mice (day 0), then 11 days after the same mice have been treated using rTSMS during 2 weeks (from day 11 to day 24). At day 25 and 40, functional recovery was analyzed using locotronic test. At day 90, mice were fixed and immunohistological analyses were performed. (B) Quantification of the number of back leg errors 25 days and 40 days after SCI. (C) Quantification of the total back leg error time 25 days and 40 days after SCI. (D) Quantification of the total crossing time 25 days and 40 days after SCI. *N* = 7 animals. Dashed lines correspond to the baseline parameters obtained during locotronic habituation (7 days before SCI). (E–G) At day 90, juvenile mice were fixed and immunohistologic analyses were performed and Stm (rTSMS treated) animals were compared to control (SCI) mice. (E, F) Representative pictures of sagittal spinal cord sections of (E) control and (F) Stm juvenile mice 90 days after SCI. Sections were stained with PDGFRβ, GFAP, and DAPI. (E–G) Fibrosis-positive area (PDGFRβ+) and quantification of PDGFRβ+ area 90 days after SCI. Scale bars are 200 μm. *N* = 5 animals per group. Quantifications are expressed as average ± SEM. * = *p* < 0.05; ** = *p* < 0.01, *** = *p* < 0.001

**Fig. 8:**
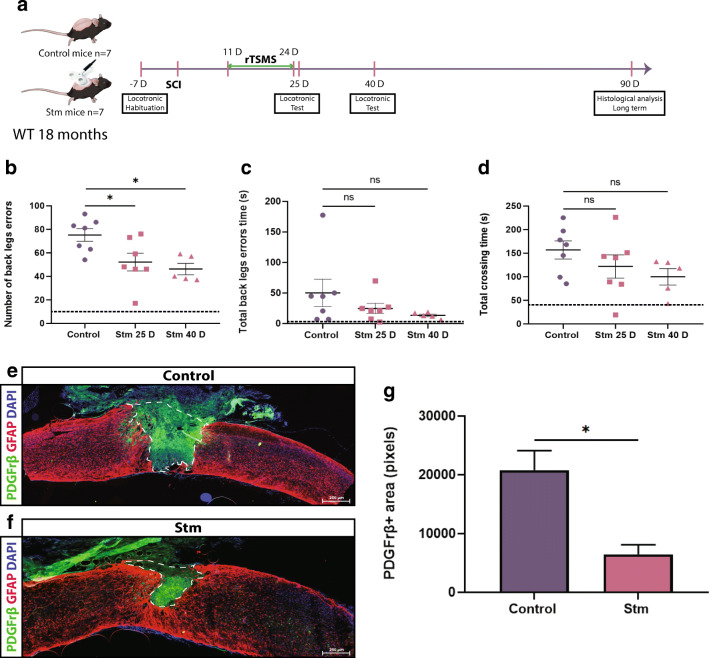
rTSMS-based treatment enhances functional recovery and modulates scar formation in a chronic condition in aged mice. (A) Experimental design, SCIs were performed on mice (day 0), then 11 days after the same mice have been treated using rTSMS during 2 weeks (from day 11 to day 24). At days 25 and 40, functional recovery was analyzed using locotronic test. At day 90, mice were fixed and immunohistological analyses were performed. (B) Quantification of the number of back leg errors 25 days and 40 days after SCI. (C) Quantification of the total back leg error time 25 days and 40 days after SCI. (D) Quantification of the total crossing time 25 and 40 after SCI. *N* = 7 animals at 25 days and *N* = 5 animals at 40 days. Dashed lines correspond to the baseline parameters obtained during locotronic habituation (7 days before SCI). (E–G) At day 90, aged mice were fixed and immunohistological analyses were performed and Stm (rTSMS treated) animals were compared to control (SCI) mice. (E, F) Representative pictures of sagittal spinal cord sections of (E) control and (F) Stm aged mice 90 days after SCI. Sections were stained with PDGFRβ, GFAP, and DAPI. (E–G) Fibrosis-positive area (PDGFRβ+) and quantification of PDGFRβ+ area 90 days after SCI. Scale bars are 200 μm. *N* = 4 animals per group. Quantifications are expressed as average ± SEM. ns = not significant * = *p* < 0.05; ** = *p* < 0.01

In Figs. 6, 7, and 8, SCIs were performed on mice (day 0), then 11 days after, the same mice have been treated using rTSMS during 2 weeks (from day 11 to day 24). At day 25 and day 40, functional recoveries were analyzed using locotronic test. At day 90, mice were fixed and immunohistological analyses were performed.

### Animal Care and Use Statement

The animal protocol was designed to minimize pain or discomfort to the animals. All experimental procedures were in accordance with the European Community guiding principles on the care and use of animals (86/609/CEE; Official Journal of the European Communities no. L358; December 18, 1986), French Decree no. 97/748 of October 19, 1987 (Journal Officiel de la République Française; October 20, 1987), and the recommendations of the Cenomexa ethics committee (#20458).

Mice of either sex were group housed (two–five mice/cage, genders separated) in secure conventional rodent facilities on a 12-h light/dark cycle with constant access to food and water.

A total of 118 mice were included in this study (Supplement Table I). We used two mouse lines in this study (100 WT C57BL/6 mice and 18 hFoxJ1-CreER^T2^ mice), including randomized grouped male and female: (1) C57BL/6 WT mice and (2) tamoxifen-inducible hFoxJ1-CreER^T2^ transgenic mice with a C57BL/6 genetic background crossed with *Rosa26-tdTomato reporter* mice. For tamoxifen-inducible mouse line, recombination was induced by five daily intraperitoneal injections of 2 mg tamoxifen (Sigma-Aldrich, 20 mg/ml in corn oil, Missouri, MO). Clearance of tamoxifen was allowed for 1 week before the start of the SCI experiments.

Experiments reported in Figs. 1, 2, 3, 4, 5, and 6 were performed on adult mice at 8–12 weeks of age (average weight 20 g for females and 25 g for males); experiments reported in Figs. 7 and 8 were performed on both: juvenile C57BL/6 mice on postnatal day (P)30 (average weight 15 g for females and 17 g for males) (Fig. 7) to correlate with teenagers and aged C57BL/6 mice at 18 months (average weight 41 g for females and 46 g for males) (Fig. 8) of age to correlate with elderly.

### Surgical Procedure and Postoperative Care

To facilitate surgical procedure, stereotaxic frame was used. Mice were kept under anesthesia with 2% of isoflurane (Iso-Vet, Osalia, Paris, France) and the animal’s body temperature has been kept steady at 37 °C with a heating pad during the entire surgical intervention. After being shaved and disinfected with 70% EtOH, the dorsal skin of the mice was incised, the superficial fat gently shifted, and the muscle tissue dissected to expose laminae T9–T11. The posterior part of vertebrae was countersank in order to create an ample space for lesion. After laminectomy, the dura mater was removed. A complete spinal cord transection was performed with 25-gauge needle for WT mice. For the transgenic mice (hFoxJ1-CreER^T2^), dorsal funiculus transaction [12] was performed in order to let the central canal uninjured. After surgery, the animals received an intramuscular injection of nalbuphine hydrochloride (10 mg/mL, Mylan, Canonsburg, PA) and were placed back in their home cages. The mice were checked daily for general health, mobility within the cage, wounds, swelling, or infections.

### rTSMS Treatment

rTSMS was delivered with a commercially available figure of eight double coil featuring an air cooling system connected to a Magstim rapid^2^ stimulator used for focal cortical and peripheral stimulations (Magstim, Whitland, UK). The coil was positioned in close contact with the back of the animal at the site of injury. The size of the area stimulated has been defined according to the manufacturer’s device manual. The area stimulated was 1.5 cm^2^. The position of the coil was maintained using an articulated arm stand. The exact position of the coil was defined using the mark located in the middle of the coil.

rTSMS treatment was applied at a frequency of 10 Hz, 10 min per day during 14 days. Stimulation protocol consisted of 10-s stimulation followed by 20 s of rest. Mice were kept under anesthesia with 2% of isoflurane during stimulation; the equivalent anesthesia were used for untreated animals. Peak magnetic intensity at the experimental distance was 0.4 T.

### Tissue Preparation and Sectioning

The animals were deeply anesthetized with sodium pentobarbital (150 mg/kg body weight) and perfused transcardially with PBS followed by ice-cold 4% formaldehyde in PBS. Dissected spinal cords were further post-fixed in 4% PFA in PBS at 4 °C overnight and cryoprotected in 30% sucrose (Life Technologies, Carlsbad, CA) for at least 48 h. After embedding in Tissue-Tek OCT compound (Sakura, Tokyo, Japan), the spinal cords were cut sagittally or axially to 20-μm thickness. Sections were collected accordingly to stereological principles (five sections per slide for sagittal sections and 10 sections per slide for axial sections) and stored at − 20 °C until further use.

### Immunohistochemistry

Spinal cord sections were blocked with 10% Normal Donkey serum (Jackson ImmunoResearch, Cambridge, UK), 0.3% Triton-X100 (Sigma-Aldrich) in PBS, then incubated overnight at room temperature in a humidified chamber with primary antibodies diluted in blocking solution. The following primary antibodies were used: Rabbit anti-Platelet-derived growth factorβ (PDGFRβ, Abcam, ab32570), Mouse anti-Glial fibrillary acidic protein (GFAP Cy3-conjugated Sigma-Aldrich, C9205 and GFAP unconjugated Sigma-Aldrich, G3893), Rabbit anti-ionized calcium-binding adapter molecule 1 (Iba1, Wako, 019-19741, Osaka, Japan), Rat anti-Myelin basic protein (MBP, Millipore, MAB386), Mouse anti-NeuN (NeuN, Millipore, MAB377), and Goat anti-Sox10 (Sox10,R&D Systems, AF2864).

After washing, antibody staining was revealed using species-specific fluorescence-conjugated secondary antibodies (Jackson ImmunoResearch). Biotinylated dextran amine (BDA, Thermo Fisher Scientific, D1956, Waltham, MA) tract-tracing was visualized with Cy3-conjugated streptavidin antibody (Jackson ImmunoResearch). Sections were counterstained with 4′,6-diamidino-2-phénylindole (DAPI; 1 μg/mL; Sigma-Aldrich) and coverslipped with Vectashield mounting media (Vector Labs, Burlingame, UK).

### Image Acquisition Analysis

The representative images of quantification for immunohistochemically stained areas for sagittal sections were taken using the Zeiss Apotome2 microscope setup. Confocal images of the axial sections were performed using the Zeiss upright fixed-stage Leica TCS SP8 CFS confocal microscope (Leica Microsystems, Wetzlar, Germany) equipped with diode laser (Coherent) at 405 nm (15%) to excite DAPI and at 552 nm (1%) to excite Tomato-Cy3, a conventional scanner at 400 Hz and a motorized stage to realize tile scan imaging. Using a 40 × (1.30, oil immersion) fluorescence, emission was sequentially detected through a hybrid detector (Leica Microsystems) in photon counting mode with a specific band from 425 to 475 nm for DAPI and 560 to 610 nm for Tomato-Cy3. The image processing and assembly was acquired with Image J software.

#### Quantification of Immunohistochemically Stained Areas

The areas of the sagittal sections were measured at the epicenter of the lesion and section rostral and caudal to the injury site; thus, a minimum of 3 sections (60 μm) per animal have been measured.

### Locotronic Test: Foot Misplacement Apparatus

Experiments have been performed as described previously by [23] (Intellibio, Nancy, France). The equipment consists of a flat ladder on which the animal can move from the starting zone towards the arrival zone. On both sides of the ladder, infrared sensors allow the visualization and recording of the displacement of the animal. The location and precise length of time of all the errors are recorded, in distinguishing the errors from front legs, back legs, and tail. Based on all data recorded, number of back leg errors, total back leg error time, and total crossing time were provided by the software and compared between groups of animals.

All the mice were pre-trained on the ladder, twice, 1 week prior to injury and then assessed on the day before the surgery in acute context to provide baseline data. For chronical context paradigm experiments (Figs. 6, 7, and 8), animals were assessed also the day before the beginning of the rTSMS treatment (10 days after SCI) to correct for any spontaneous recovery (Supplement Fig I).

### Proteomic Analyses

The experimental design for proteomics analyses was the same as that for immunohistological experiments. SCIs were performed on mice (day 0), then half of the mice has been treated using rTSMS during 2 weeks (from day 1 to day 14). At day 15, mice were perfused with cold PBS to remove the blood, then spinal cords (1 cm) were dissected on ice and directly frozen in tube at − 80 °C.

#### Protein Extraction

Seven hundred microliters of solubilization buffer (urea 7 M, Sigma-Aldrich, thiourea 2 M, Sigma-Aldrich, DTT 20 mM, Sigma-Aldrich, CHAPS 2%, Sigma-Aldrich, C7BzO 0.5%, Sigma-Aldrich, Tributylphosphin 5 μL, FlukaChemika, Zurich, Switzerland) was added to each sample in Lysing Matrix D (Biorad, Hercules, CA). Samples were homogenized in the FastPrep® machine for 40 s at a speed of 6 m/s, after which they were centrifuged at 10000*g* for 8 min. This operation was repeated 3 times to grind the entire tissue. Supernatants were transferred to a clean 1.5-mL microcentrifuge tube and conserved at − 80 °C.

#### Protein Concentration

To estimate the injury site protein concentration for each extract, Bradford was performed according to the manufacturer’s instructions (Biorad). The linear range for BSA standard was 0.1 to 1 mg/mL. The dilution of all samples was 1:20. Solubilization buffer was used as a negative control. Absorbance at 595 nm was measured with the Perkin Elmer Wallac 1420 Victor3™.

#### Enzymatic Digestion of Protein Extracts

For each extract, 25 μg of proteins was loaded into a 7% polyacrylamide gel (Acrylamide/Bis-Acrylamide 30% [29:1], Sigma-Aldrich) and a migration was performed for a short period (90 min at 10–20 mA/gel). After Coomassie blue staining, the revealed protein bands were excised and immersed for the first time in a reductive buffer (5 mM DTT, Sigma-Aldrich), and in a second time, in an alkylated buffer (20 mM iodoacetamide, Sigma-Aldrich). After washing steps, gel bands were submitted to protein digestion by 1 μg of trypsin (Promega, Madison, WI). After overnight incubation at 37 °C, several steps of peptide extraction were performed using acetonitrile (Fisher, Hampton, NH). Then, the peptide fractions were combined and dried.

#### NanoLC-MS/MS Analyses

All experiments were performed on an LTQ-Orbitrap Elite apparatus coupled to an Easy nLC II system (both from Thermo Scientific). Samples were injected onto an enrichment column (Acclaim PepMap100, Thermo Scientific). The separation was achieved with an analytical column needle (NTCC-360/100-5-153, Nikkyo Technos, Tokyo, Japan). The mobile phase consisted of H_2_O/FA 0.1% (buffer A) and CH_3_CN/FA 0.1% (buffer B). Tryptic peptides were eluted at a flow rate of 300 nL/min using a three-step linear gradient: from 2 to 40% B over 75 min, from 40 to 80% B in 4 min, and at 80% B for 11 min. The mass spectrometer was operated in positive ionization mode with capillary voltage and source temperature set at 1.5 kV and 275 °C, respectively. The samples were analyzed using CID (collision-induced dissociation) method. The first scan (MS spectra) was recorded in the Orbitrap analyzer (*R* = 60,000) with the mass range *m*/*z* 400–1800. Then, the 20 most intense ions were selected for MS2 experiments. Singly charged species were excluded for MS2 experiments. Dynamic exclusion of already fragmented precursor ions was applied for 30 s, with a repeat count of two, a repeat duration of 30 s, and an exclusion mass width of ± 5 ppm. The precursor isolation width was 2 *m*/*z*. Fragmentation occurred in the linear ion trap analyzer with normalized collision energy of 35. All measurements in the Orbitrap analyzer were performed with on-the-fly internal recalibration (lock mass) at *m*/*z* 445.12002 (polydimethylcyclosiloxane).

#### Database Searches

Raw data files were processed using Proteome Discoverer 1.4 software (Thermo Scientific). Peak lists were searched using the Mascot search software (Matrix Science, version 2.2.04) against the database Uniprot with the taxonomy *Mus musculus*. Database searches were performed with the following parameters: two missed trypsin cleavage sites allowed; variable modifications: carbamidomethylation on cysteine, and oxidation on methionine. The parent-ion and daughter-ion tolerances were 10 ppm and 0.5 Da, respectively. False discovery rate (FDR) threshold for identifications was specified at 1% (for proteins and peptides).

#### Quantification

For protein quantification, a label-free experiment was performed as previously described by Kentache et al. [24]. Briefly, after MS analysis, raw data were imported in Progenesis LC-MS software (Nonlinear Dynamics, version 4.0.4441.29989, Newcastle, UK). For comparison, one sample was set as a reference and the retention times of all other samples within the experiment were aligned. After alignment and normalization, statistical analysis was performed for one-way ANOVA calculations. For quantitation, peptide features presenting a *p* value and a *q*-value less than 0.05, and a power greater than 0.8, were retained. MS/MS spectra from selected peptides were exported for peptide identification with Mascot (Matrix Science, version 2.2.04). Database searches were performed with the following parameters: 1 missed trypsin cleavage site allowed; variable modifications: carbamidomethylation of cysteine and oxidation of methionine. Mass tolerances for precursor and fragment ions were set at 5 ppm and 0.35 Da, respectively. False discovery rates were calculated using a decoy-fusion approach in Mascot (version 2.2.04). Identified peptide-spectrum matches with − 10logP value of 13 or higher were kept, at a FDR threshold of 5%. Mascot search results were imported into Progenesis. For each growth condition, the total cumulative abundance of the protein was calculated by summing the abundances of peptides. Proteins identified with less than 2 peptides were discarded.

### BDA Tract-Tracing

Tract-tracing of propriospinal neurons was performed by injection of BDA, 10% wt/vol in sterile saline injected 1 μl, 4-mm rostral to the injury site, 2 days after SCI (Fig. 4H) as described by [9].

### Neural Stem Cells Cultures

Animals were sacrificed 15 days after SCI. Spinal cord cells were dissociated and neurosphere cultures were established as described [25, 26]. All cells isolated from one spinal cord were plated in 75-cm^2^ culture dishes. First, neurospheres were harvested after 2 weeks in culture and then were dissociated into single cells for passage. Approximately 100,000 cells per animal were plated in a 25-cm^2^ culture dish for the next generation of neurospheres, and all the new neurospheres (second, third, and fourth generations) were harvested after 1 week in culture.

### Quantification of Ependymal Cell Reactivity and Contribution to the Glial Scar

To quantify ependymal cell reactivity and contribution to the glial scar, the central canal and the dorsal part of the spinal cord have been defined. To do so, we used a characterized transgenic mouse line hFoxJ1-CreERT::tdTomato that expresses tdTomato in ependymal and ependymal-derived cell bodies allowing the quantification of mean fluorescence intensity of tomato+ cells between the control and Stm groups. A tile software was used to create images of axial sections at the epicenter of the lesion, then measurements of immunoreactivity in the defined areas were quantified using Image J software.

### Statistical Analysis

All data are presented as means ± standard error of the mean (SEM). Comparison of means were performed using two-tailed Mann-Whitney test for all the experiments except for proteomic analysis where one-way ANOVA test has been used. In all tests, *p* < 0.05 were considered statically significant. Supplement Table II lists all tests performed, the comparison made, and the associated *p* values.

## Results

### rTSMS-Based Treatment Modulates Spinal Scar Formation After SCI

To investigate whether rTSMS can promote scar formation in both acute and chronic phases following SCI, we performed spinal cord transection in adult mice [27]. One day after SCI, mice were treated by rTSMS for 14 consecutive days, and were sacrificed on day 15 or 90 for analysis (Fig. 1A). As the lesion site can be subdivided into a lesion core infiltrated by mostly stromal-derived pericytes and the boarder where reactive astrocytes accumulate, we performed immunohistochemistry for these areas by PDGFRβ and GFAP markers respectively. Our data revealed that the PDGFRβ+ area is significantly reduced by 2 and 4 times in the rTSMS treatment group compared to control, 15 days, and 90 days after SCI respectively (Fig. 1B–D, E–G respectively). Similarly, we compared the core of glial scar by GFAP-negative area and showed substantial reduction in the rTSMS animals in both short- and long-term conditions (Fig. 1H–J, K–M respectively). Additional comparisons have been performed in order to investigate the evaluation of the scar overtime. It appears that there is no significant difference between 15 and 90 days after SCI for PDGFRβ+ areas (Supplement Fig IIA) or GFAP-negative areas (Supplement Fig IIB) in both groups of animals (control and Stm groups) (Supplement Fig II).

In addition, we investigated Iba1 expression; Iba1 is a microglial and macrophage-specific marker involved with the membrane ruffling and phagocytosis in activated microglia. Our results showed that Iba1+ area in the rTSMS group is also significantly decreased by nearly 3 times (Fig. 1N–P), indicating that rTSMS treatment modules spinal scar by reducing fibrosis and inflammation and by increasing astrocytic scar formation (GFAP+, quantified by GFAP-negative area). Moreover, rTSMS beneficial effects on scar formation are sustained even several months after the end of the treatment.

### rTSMS-Based Treatment Enhances Functional Recovery

To further investigate whether rTSMS treatment can promote functional recovery, we conducted sensorimotor tests using a foot misplacement apparatus (Fig. 2A) [23]. We showed that the numbers of back leg errors and total back leg error time have already exhibited reduction trends (Fig. 2B, C) and the total crossing time was significantly reduced since 15 days after SCI in the rTSMS group (Fig. 2D) (see Movie SI and Movie SII). Moreover, all these sensorimotor parameters were significantly improved 30 days after SCI in the rTSMS group (Fig. 2E–G).

### rTSMS Regulates Functional Protein Synthesis and Pathways

We described above that 14 days of rTSMS treatment favor tissue repair and functional recovery after SCI in mice. To gain insight on molecular basis of recovery, a proteomic analysis was designed to highlight the regulation of biological functions occurring under this stimulation. Accordingly, spinal cord tissues (1 cm) from stimulated and control mice were collected (*n* = 4 for both groups). After protein extraction, trypsin proteolysis was performed. Then, the resulting complex peptide mixtures were analyzed by nanoLC-MS/MS. Finally, from MS data, bioinformatics tools were used to quantify protein amounts, and then to analyze the pathways involved (Fig. 3A).

Differential proteomic analysis first revealed that the intra-group peptide ion intensities (nearly 50,000 distinct quantitative data) were associated with a good reproducibility (averaged regression coefficient of 0.91 for both groups). After quantitative analysis and identification, 156 proteins appeared differentially regulated between rTSMS-treated and control mice (Supplement Table III). Among them, 148 proteins were upregulated and 8 were downregulated in stimulated mice. As a whole, the abundance ratios were rather limited, although associated to statistical significance (Supplement Table III). All of these proteins were then integrated as inputs for functional analysis (IPA, Qiagen). It allowed to describe 37 biological functions increased in stimulated mice (*z*-score > 2) and 7 decreased (*z*-score < − 2). Among those, some were of particular interest in the context of this study, especially regarding both neural development and cell death (Fig. 3B). Thus, 62 and 26 proteins displaying a decreased abundance were involved in apoptosis and in neuronal cell death, respectively. Conversely, numerous functional pathways were promoted, namely development of neurons, branching and proliferation of neural cells, and axonogenesis. These functional regulatory effects are summarized in Fig. 3C, with a chart showing development of a complex protein network leading to neuronal survival after rTSMS treatment.

Although altogether all these single observations were highly convergent, we performed additional validation experiments by immunohistochemistry targeted on the above-described pathways.

### rTSMS Inhibits Demyelination and Enhances Neuronal Survival and Axonal Regrowth

To further validate our proteomic data, we evaluated myelination, axon regeneration, and neuronal death in rTSMS and control mice 15 days after SCI (Fig. 1A). We first analyzed the demyelination after SCI by using MBP staining in WT mice. We found that non-myelinating area (MBP-negative area) is reduced by almost 4 times in rTSMS-treated group following SCI (Fig. 4A–C). We further investigated the effects of rTSMS treatment on neuronal survival using NeuN-positive cell quantification in hFoxJ1-CreERT::tdTomato mice. We quantified axial sections 40 μm rostrally or caudally to the epicenter, and showed that average survived neurons significantly increased from 139/section (control) to 233/section (rTSMS) (Fig. 4D–F). To further study whether there is also better axonal regrowth or axon survival in rTSMS-treated group, we performed BDA tract-tracing experiments (Fig. 4G–H). After injecting BDA dye 2 days following SCI in both groups, we performed stimulations in the rTSMS group for 2 weeks and sacrificed all the mice on the 16th day for longitudinal sectioning and analysis (Fig. 4G–H). Our data showed that there is a substantial infiltration of BDA+ axons into the lesion site, while very few signals are shown in the control group, suggesting that rTSMS treatment induces axonal regrowth/axon survival into the lesion core (Fig. 4I, J). Altogether, these results show that rTSMS decreases demyelination and increases neuronal survival and axonal regrowth/axon survival.

### rTSMS Increases Proliferation and Modulates Differentiation of Spinal Cord Stem Cells

As our proteomic data suggest that rTSMS promotes development of neurons and proliferation of neural cells (Fig. 3), we wonder whether spinal cord neural stem cells can be activated by rTSMS and contribute to regeneration. Therefore, we used both in vitro neurosphere assays and in vivo analysis to study ependymal cells and spinal cord stem cells, and to analyze their reactivity following SCI (Fig. 5A) [10–12]. First, we sacrificed the mice from control and rTSMS, 15 days after SCI, and performed neurosphere assay to study the self-renewal potential of ependymal cells. We found that rTSMS treatment increases the self-renewal potential of spinal cord stem cells by 2-fold in vitro (Fig. 5B). To further study ependymal cells’ reactivity in vivo, we took advantage of the inducible transgenic hFoxJ1-CreERT::tdTomato mouse line [25]. Ependymal cell reactivity was evaluated around the central canal and in the lesion site by quantifying mean fluorescence intensity of tdTomato-positive cells (Fig. 5C). This reveals that rTSMS increases reactivity of spinal cord stem cells and their contribution to the scar (Fig. 5D, E).

Then, we investigated the effects of rTSMS on the fate of ependymal-derived cells after SCI (Fig. 5F–K). Fifteen days after SCI, we observed that the ependymal progeny gives rise to 9% of GFAP+ cells in the control group; in comparison, we found that there was almost two times more Tomato+GFAP+ cells (15%) in the rTSMS group (Fig. 5F–H). Moreover, based on Sox10 staining, we observed that there is a significant increase in the number of Tomato+Sox10+ cells (7.5%) in the rTSMS group in comparison to the control group (3.5%) (Fig. 5I–K).

These results indicate that rTSMS induces more ependymal cells to differentiate into astrocytes and oligodendrocytes.

### rTSMS-Based Treatment Enhances Functional Recovery in a Chronic Condition

Acute rTSMS stimulation (the day following SCI) cannot be used for most SCI-affected patient following traffic accidents, falls, and violence [28]. From the therapeutic perspective, we thus tested rTSMS in a chronic condition. We began rTSMS treatment 10 days after SCI for a 14-day period as described above (Fig. 6A). We measured functional recoveries and tissue repair on the same animals throughout the experiments. Our results show that 25 days and 40 days after SCI, rTSMS treatment in this paradigm significantly enhances sensorimotor recoveries, including back leg errors (5 times reduction), back leg error time (10 times reduction), and total crossing time (2 times reduction) (Fig. 6B–D). On these animals, histological analyses reveal that rTSMS modulates scar formation by decreasing the fibrotic (PDGFRβ+) and increasing the astrocytic (GFAP+, quantified by GFAP-negative area) scar 90 days after SCI (Fig. 6E–G, H–J respectively).

### rTSMS-Based Treatment Enhances Functional Recovery and Modulates Scar Formation in a Chronic Condition in Juvenile and Aged Mice

Epidemiology of the SCI evolved overtime; currently, in developed countries, two categories of persons are more concerned by SCI, teenagers and the elderly [17]. We further investigate whether this rTSMS treatment is applicable in SCI animals at different age. Using the same study design (Figs. 7A and 8A), we tested the effects of rTSMS on juvenile (P30) (Fig. 7) and aged (18 months) (Fig. 8) animals.

In juvenile mice, locomotor experiments show that rTSMS treatment induces sensorimotor recovery 25 days and 40 days after SCI (Fig. 7B–D). On these animals, histological analyses reveal that rTSMS treatment reduces the fibrotic scar 90 days after SCI (Fig. 7E–G).

In aged mice, locomotor experiments show that rTSMS treatment induces a moderate sensorimotor recovery 25 days and 40 days after SCI (Fig. 8B–D). Indeed, for all parameters tested, only the number of back leg errors is significantly improved 25 and 40 days after SCI (Fig. 8B). On the other hand, as previously described for adult and juvenile animals, histological analyses reveal that rTSMS treatment reduces the fibrotic scar 90 days after SCI (Fig. 8E–G).

## Discussion

rTMS is a safe and noninvasive form of CNS stimulation that applies focal magnetic field to generate electric currents [29]. rTMS can modulate CNS activity depending on frequency and coil position [30, 31].

This therapy is being evaluated in a number of clinical contexts for the treatment of stroke [32], tinnitus [33], and major depressive disorders [14], as well as for the management of neurodegenerative disorders such as Parkinson’s [34] or Alzheimer’s diseases [17].

Different studies described the applications of noninvasive neuromodulatory techniques after SCI such as transcutaneous electric stimulation [35], direct current stimulation [36], or magnetic stimulation [37]; all are new therapeutic tools which can be used for experimental and clinical research. Both transcutaneous electric stimulation and direct current stimulation are designed to mainly stimulate individual group of neurons, whereas figure of eight double coil–based TMS delivers a focal magnetic field stimulating neural tissue structure (0.5 mm^2^ to 2 cm^2^ according to the size of the coils) [38, 39]. In our study, one of the main objectives was to modulate noninvasively the lesion site after SCI that is why rTSMS has been chosen.

For SCI, very few experimental and clinical investigations have explored the benefit of the rTMS following SCI, by using full-body or brain stimulation [40–43]. However, full-body stimulations may activate cells from other regions, such as the brain, and therefore might have an impact on the specificity of the rTMS effects on the spinal cord. Moreover, the cellular and the molecular changes that underpin the effect outcomes are largely unknown.

Although some previously published studies described the use of rTSMS after SCI, their stimulation coils and protocols are different from ours. Indeed, the study by Petrosyan et al. explores the use of focal rTSMS after SCI on a rat model [44]. However, the protocol is based on a low frequency (0.2 Hz) and very high intensity (2.8 T) stimulation during 5 weeks. Moreover, the protocol was composed of one train of stimulation during 35 min. More importantly, in this study, there was no difference between rTSMS treated and untreated animals. Another study by Leydeker et al. used a non-focal stimulation protocol. Indeed, they used a circular coil of 5 cm which implied full-body stimulations [42].

Therefore, we used a focal rTSMS paradigm as treatment after SCI, to avoid side effects. We explored the feasibility of the rTSMS, evaluated its effects on tissue repair and functional recoveries, and we characterized the cellular and molecular mechanisms induced by rTSMS after SCI. As a proof of concept, we used our established rTSMS protocol at a frequency of 10 Hz, 10 min per day for 2 weeks. We chose these parameters in accordance with other protocols previously described [22, 45].

SCI causes massive cell death and inflammation, leading to free radical and cytokine production which exacerbates neurons and oligodendrocyte apoptosis at the lesion site and axonal degeneration above the injury [46, 47]. Following SCI, a scar is formed which is composed of a fibrotic and a glial component. The fibrotic component is constituted by a subset of perivascular cells, termed type A pericytes, which creates a scar core of fibroblast-like cells and dense extracellular matrix [5, 10]. Newly, Dias et al. report that significantly more descending axons can grow through the lesion site after SCI when this fibrotic lesion core is reduced [7]. On the other hand, the glial component is generated by astrocytes derived from resident astrocytes and by ependymal cell–derived astrocytes [11, 25]. The resident astrocytes increase their proliferation after SCI and upregulate the expression of GFAP near the lesion site and in the surrounding area. The glial component prevents inflammation and immune cells and fibroblast-like cell infiltration. Recently, by using transgenic loss-of-function mouse model to ablate scar-forming astrocytes, it has been demonstrated that astrocytes express axon-growth-supporting molecules which aid axonal regeneration after SCI [6].

These studies suggest the fibrotic and astrocyte scar modulation as a potential target to promote axonal regeneration after SCI. In this regard, we investigated if rTSMS can modulate fibrotic and astrocyte scars upon SCI. Our results show that rTSMS has the ability to modulate scar formation 15 days after SCI and that these effects are sustained 90 days after SCI. In fact, similar to what was described in previous studies, fibrotic scar represents a major barrier for axonal regrowth and inhibition of it using rTSMS increases in turn astrogliosis and decreases inflammation [6, 10, 48].

We further investigated if rTSMS treatment can promote functional recovery. To this purpose, we used a foot misplacement apparatus (locotronic test) and we demonstrated that rTSMS-based treatment enhances sensorimotor functions after SCI by decreasing the number of back leg errors, total back leg error time, and total crossing time. These results are in agreement with a previously published study in rats showing that magnetic field promotes the restoration of locomotor function after SCI [43].

Then, to obtain more profound molecular knowledge about these effects, we performed proteomic analysis which revealed that 156 proteins are differentially regulated after rTSMS. Ingenuity Pathway Analysis of these proteins shows that rTSMS increased 37 biological functions and decreased 7 functions. Within these candidates, the targets for improving neurological recovery following SCI are mostly directed to limit the diverse cellular, molecular, and biochemical changes that self-destruct spinal cord tissue and impede neurological recovery following SCI. To this end, we focused on 6 key biological processes modulated after rTSMS which are directly related to these secondary injury events. We found 4 biological processes underwent upregulation upon stimulation: (1) proliferation of neural cells, (2) axonogenesis, (3) branching of neurons, and (4) growth of axons. Among these 4 families, we validated them in 2 aspects: (a) axonal regrowth and neuronal branching; (b) neural cell proliferation. Interestingly, proteomic analysis reveals that a high number of myelin and microtubule proteins is upregulated in stimulated group such as MBP, MAPT, MOG, CNP, and Nefm.

It is known that myelination is regulated by many intrinsic and extrinsic factors like neuronal activity [49, 50]. It can be hypothesized that rTSMS have a cell extrinsic role via neuronal activity such as promoting the maturation of oligodendrocyte precursor cells or their survival and enhances their myelination potential [19, 51] which could be the cause underlying our findings.

Proteomic analysis also reveals that rTSMS upregulates proteins related to neural cell proliferation and cell migration such as SYN1, NCDN, GAP43, PAFAH1, and RHOG*.* Likewise, we found two important biological functions downregulated after stimulation in accordance with previous functions—apoptosis and neural cell death—with expression of specific proteins including SOD1/2, PRDX, MAPK3, Cdc42, and SNCA.

In order to validate our proteomic analysis results, we investigated the effect of rTSMS on neuronal survival, axonal regeneration, and remyelination. Indeed, after injury, the axonal skeleton disintegrates, and the axonal membrane breaks, which leads to axonal degeneration and release of myelin debris, one of the main causes of axonal regeneration inhibition [46, 52]. This accumulation of myelin debris in turn leads to the apoptosis of neurons and oligodendrocytes, and further contributes to the failure of remyelination and regeneration [53]. We report that rTSMS after SCI decreases the demyelination process and enhances neuronal survival objectified respectively by the reduction of the MBP-negative area and by the augmentation of the number of NeuN-positive cells. Then, we performed BDA tract-tracing experiments to analyze axonal regrowth/axon survival because tract-tracing is considered the gold standard for studying new growth from axonal systems [54]. It appears that rTSMS treatment induces axonal regrowth/axon survival into the lesion core compared to control (SCI) animals.

Since the tissue regeneration promoted by rTSMS is also partly due to activation of neural cell proliferation, suggested by the proteomics, we further investigate the effects of rTSMS on stem cell proliferation.

Ependymal cells, astrocytes, and OPC are highly proliferative after SCI and display different cellular responses [55, 56]. Each glial cell type has the ability to respond to electrical activity directly or indirectly making them likely cellular effectors of rTSMS [20].

During adulthood, the spinal cord stem cell potential is restricted to ependymal cells. Indeed, we and others previously showed that ependymal cells are the only neural stem cell source in the spinal cord since early postnatal stage throughout adulthood [12]. Moreover, ependymal cells can be activated by SCI, by showing high proliferation, glial differentiation, and massive migration to the injury site for scar formation, as well as secretion of neurotrophic factors for neuronal survival [10, 11]. First, to investigate the effects of rTSMS on ependymal cell reactivity after SCI, we took advantage of the inducible hFoxJ1-CreERT::tdTomato mouse line, to specifically fate map ependymal cells. Our results show that rTSMS after SCI can enhance proliferation of ependymal cells in vitro and their reactivity in vivo compared to the control group. In a second time, we studied the effects of rTSMS on the progeny of ependymal cells. To do so, we analyzed ependymal-derived cells 15 days after SCI. Our data demonstrate that rTSMS enhances differentiation of ependymal cells to oligodendrocyte and astrocyte lineages.

Our results are in line with the finding of a previous study that assessed the effects of rTMS on neural stem cell and progenitor cell (NS/PCs) proliferation and differentiation. Indeed, Abbasnia et al. showed that rTMS increases the number and the size of the neurospheres obtained from adult mouse subventricular zone [57].

Our findings have strengthened the theories from previous studies that rTMS influences a wide variety of cells without direct activations of neuronal firing [58] including glial cells [20]. Indeed, Cullen et al. showed that rTMS increases the number of newborn oligodendrocytes in the adult mouse cortex and their survival and enhances myelination [51]. Moreover, several studies demonstrate that rTMS increase cell differentiation and proliferation; Guo et al. indicate that the application of 10-Hz rTMS significantly increases the proliferation of adult neural stem cells (NSCs) in the subventricular zone [52]. Menghu et al. showed that rTMS after intracerebral hemorrhage in a mouse model enhances the proliferation and neuronal differentiation of NSCs through the MAPK signaling pathway [59]. It has been also shown that rTMS decreases apoptosis; indeed, Gao et al., using a mouse model of transient ischemia, report that rTMS reduces the number of caspase-3-positive cells [18].

Finally, it is well described that rTMS influences neuronal functions; in particular, it increases their neurotrophic factors production and release. Several studies described that rTMS enhances BDNF and VEGF expression [21, 60].

Lastly, we investigated whether rTSMS can be applied in a more clinical-related context, when the treatment can only begin 10 days after SCI. Indeed, in most of the cases, SCI is due to traffic accidents; these patients could beneficiated of the rTSMS-based treatment only when they are stabilized and there is no longer any vital issue. Mainly, these patients stay 10–14 days in the intensive care unit after their accident; that is why we have tested rTSMS during 14 consecutive days, starting the treatment 10 days after SCI. In this paradigm, it appears that rTSMS induced functional recovery and tissue repair in adult mice. Moreover, the incidence of the SCI is higher in two populations of patients, the teenagers and the elderly [61]. We tested whether rTSMS can be potentially applied on patients within these categories. Our results demonstrate that rTSMS induce functional recovery and tissue repair in both juvenile and aged mice. We note in accord with the literature that aged mice have considerably less efficient outcomes than juvenile mice probably due to age-related physiological changes [62]*.* To further investigate the respective effects of SCI and rTSMS according to the age of mice, we performed additional comparisons of the locomotor performance between adult and juvenile mice and between adult and aged mice (Supplement Fig III). These analyses compare the data obtained 10 days after SCI (1 day before the beginning of rTSMS treatment—control) and 25 and 40 days after SCI (15 days and 30 days respectively after the beginning of rTSMS treatment). Our results show that there is no main difference 10 days after SCI (Control) between adult and juvenile mice (Supplement Fig IIIA-C), except for the total back leg error time. More interestingly, it appears that there is no difference 25 and 40 days after SCI between adult and juvenile mice (Supplement Fig IIIA-C). These results suggest that SCI and also rTSMS have the same impact on locomotor functions in adult and juvenile mice. In the same way, our results show that there is no main difference 10 days after SCI (control) between adult and aged mice (Supplement Fig IIID-F), except for the total crossing time, suggesting that SCI induces the same motor deficits in both adult and aged mice. At the opposite, it appears that 25 and 40 days after SCI, the locomotor functions are much worse in aged mice in comparison to adult ones (Supplement Fig IIID-F), indicating that rTSMS induces better functional recovery in adult mice in comparison to aged mice.

Mice models of rTSMS with different paradigms at different ages play a crucial role in aiding the understanding of the cellular and molecular mechanisms underlying rTSMS effects after SCI. However, one of the main limitations to rodent models of rTMS is the lack of rodent-specific TMS stimulator coils [63]. In all study including ours, commercial human coils which are large are used, inducing a lack of focality. In our study, the area stimulated was 1.5 cm^*2*^, meaning that 3–4 vertebral segments have been stimulated. For a better translation to clinical, there is a need to develop a rodent-specific coil that can deliver rTMS at high intensity while maintaining a good degree of focality.

Various animal and SCI models have been employed to understand the pathophysiology of SCI and to develop therapeutic strategies [64]. In our study, we used a complete transection model due to the fact that it is highly reproducible and allow to specifically study axonal regeneration and recovery of locomotion [27]. However, to a clinical point of view, this model is less relevant. Indeed, clinically, the most common form of injury is a contusion injury. Nevertheless, the cellular responses which follow injury are similar between contusion and transection [65]. Indeed, after contusion, the scar is composed of a fibrotic scar associated with hematogenous macrophages and of an astroglial scar occupied by microglia [66]. Moreover, it is described that after SCI, mice show some regeneration and spontaneous recovery [67], in particular after contusive injury. In our study, we performed a complete transection; as shown in Supplement Fig III, there is no tissue repair in control mice which induces physical deterioration overtime in this group (Fig. 2).

## Conclusion

Altogether, our findings report that rTSMS induces therapeutic effects in juvenile, adult, and aged rodent models following SCI by modulating the scar formation, increasing proliferation and differentiation of spinal cord stem cells to glial cells, and promoting remyelination, which lead to axonal regrowth, neuronal survival, and locomotor recovery. Our result suggests that rTSMS could be a promising therapy following SCI and pave the way for preclinical translation.

## Electronic Supplementary Material

ESM 1 (PDF 473 kb)

ESM 2 (MP4 13,250 kb)

ESM 3 (MP4 14,053 kb)

ESM 4 (PDF 490 kb)

ESM 5 (PDF 490 kb)

## Data Availability

The authors confirm that the data supporting the findings of this study are available within the article and its Supplement material.

## Abbreviations

BDA: biotinylated dextran amine
DAPI: 4′,6-diamidino-2-phénylindole
GFAP: glial fibrillary acidic protein
Iba1: ionized calcium-binding adapter molecule 1
MBP: myelin basic protein
PDGFRβ: platelet-derived growth factorβ
rMS: repetitive magnetic stimulation
rTSMS: repetitive trans-spinal magnetic stimulation
SCI: spinal cord injury
WT: wild type

## References

[1] Wilson JR, Cadotte DW, Fehlings MG (2012). Clinical predictors of neurological outcome, functional status, and survival after traumatic spinal cord injury: a systematic review. J Neurosurg Spine..

[2] Siddiqui AM, Khazaei M, Fehlings MG (2015). Translating mechanisms of neuroprotection, regeneration, and repair to treatment of spinal cord injury. Progress in Brain Research.

[3] Tran A (2018). The Biology of Regeneration Failure and Success After Spinal Cord Injury. Physiol Rev..

[4] Cregg JM, DePaul MA, Filous AR (2014). Functional regeneration beyond the glial scar. Experimental Neurology..

[5] Goritz C, Dias DO, Tomilin N (2011). A Pericyte Origin of Spinal Cord Scar Tissue. Science..

[6] Anderson MA, Burda JE, Ren Y (2016). Astrocyte scar formation aids central nervous system axon regeneration. Nature..

[7] Dias DO, Kim H, Holl D (2018). Reducing Pericyte-Derived Scarring Promotes Recovery after Spinal Cord Injury. Cell.

[8] Dias DO, Göritz C (2018). Fibrotic scarring following lesions to the central nervous system. Matrix Biol..

[9] Anderson MA, O’Shea TM, Burda JE (2018). Required growth facilitators propel axon regeneration across complete spinal cord injury. Nature..

[10] Sabelström H, Stenudd M, Réu P (2013). Resident neural stem cells restrict tissue damage and neuronal loss after spinal cord injury in mice. Science..

[11] Barnabé-Heider F, Göritz C, Sabelström H (2010). Origin of new glial cells in intact and injured adult spinal cord. Cell Stem Cell..

[12] Li X, Floriddia EM, Toskas K (2016). Regenerative Potential of Ependymal Cells for Spinal Cord Injuries Over Time. EBioMedicine..

[13] Garbossa D, Boido M, Fontanella M (2012). Recent therapeutic strategies for spinal cord injury treatment: possible role of stem cells. Neurosurg Rev..

[14] Becker JE, Shultz EKB, Maley CT (2019). Transcranial Magnetic Stimulation in Conditions Other than Major Depressive Disorder. Child Adolesc Psychiatr Clin N Am..

[15] Khedr EM, Etraby AE, Hemeda M (2010). Long-term effect of repetitive transcranial magnetic stimulation on motor function recovery after acute ischemic stroke. Acta Neurologica Scandinavica..

[16] Ba F, Zhou Y, Zhou J (2019). Repetitive transcranial magnetic stimulation protects mice against 6-OHDA-induced Parkinson’s disease symptoms by regulating brain amyloid β1-42 level. Mol Cell Biochem..

[17] Rabey JM, Dobronevsky E (2016). Repetitive transcranial magnetic stimulation (rTMS) combined with cognitive training is a safe and effective modality for the treatment of Alzheimer’s disease: clinical experience. J Neural Transm.

[18] Gao F, Wang S, Guo Y (2010). Protective effects of repetitive transcranial magnetic stimulation in a rat model of transient cerebral ischaemia: a microPET study. Eur J Nucl Med Mol Imaging..

[19] Prasad A, Teh DBL, Blasiak A (2017). Static Magnetic Field Stimulation Enhances Oligodendrocyte Differentiation and Secretion of Neurotrophic Factors. Scientific Reports..

[20] Cullen CL, Young KM (2016). How Does Transcranial Magnetic Stimulation Influence Glial Cells in the Central Nervous System?. Frontiers in Neural Circuits.

[21] Grehl S, Viola HM, Fuller-Carter PI (2015). Cellular and Molecular Changes to Cortical Neurons Following Low Intensity Repetitive Magnetic Stimulation at Different Frequencies. Brain Stimulation..

[22] Makowiecki K, Harvey AR, Sherrard RM (2014). Low-intensity repetitive transcranial magnetic stimulation improves abnormal visual cortical circuit topography and upregulates BDNF in mice. J Neurosci..

[23] Chort A, Alves S, Marinello M (2013). Interferon β induces clearance of mutant ataxin 7 and improves locomotion in SCA7 knock-in mice. Brain..

[24] Kentache T, Abdelkrim AB, Jouenne T (2017). Global Dynamic Proteome Study of a Pellicle-forming Acinetobacter baumannii Strain. Molecular & Cellular Proteomics..

[25] Meletis K, Barnabé-Heider F, Carlén M (2008). Spinal cord injury reveals multilineage differentiation of ependymal cells. PLoS Biol..

[26] Li X, Floriddia EM, Toskas K (2018). FoxJ1 regulates spinal cord development and is required for the maintenance of spinal cord stem cell potential. Experimental Cell Research..

[27] Cheriyan T, Ryan DJ, Weinreb JH (2014). Spinal cord injury models: a review. Spinal Cord..

[28] Weidner N, Rupp R, Tansey K, editors. Neurological Aspects of Spinal Cord Injury. Springer International Publishing; 2017.

[29] Barker AT, Jalinous R, Freeston IL (1985). Non-invasive magnetic stimulation of human motor cortex. Lancet..

[30] Hoppenrath K, Härtig W, Funke K (2016). Intermittent Theta-Burst Transcranial Magnetic Stimulation Alters Electrical Properties of Fast-Spiking Neocortical Interneurons in an Age-Dependent Fashion. Front Neural Circuits..

[31] Tang A, Thickbroom G, Rodger J (2017). Repetitive Transcranial Magnetic Stimulation of the Brain: Mechanisms from Animal and Experimental Models. Neuroscientist..

[32] Dionísio A, Duarte IC, Patrício M (2018). The Use of Repetitive Transcranial Magnetic Stimulation for Stroke Rehabilitation: A Systematic Review. J Stroke Cerebrovasc Dis..

[33] Mulders WHAM, Vooys V, Makowiecki K, et al. The effects of repetitive transcranial magnetic stimulation in an animal model of tinnitus. Scientific Reports. 2016 ;6. 10.1038/srep38234 PMC5131273 27905540

[34] Torres F, Villalon E, Poblete P (2015). Retrospective Evaluation of Deep Transcranial Magnetic Stimulation as Add-On Treatment for Parkinson’s Disease. Front Neurol..

[35] Rejc E, Angeli CA (2019). Spinal Cord Epidural Stimulation for Lower Limb Motor Function Recovery in Individuals with Motor Complete Spinal Cord Injury. Phys Med Rehabil Clin N Am..

[36] Song W, Martin JH (2017). Spinal cord direct current stimulation differentially modulates neuronal activity in the dorsal and ventral spinal cord. J Neurophysiol..

[37] Hunanyan AS, Petrosyan HA, Alessi V (2012). Repetitive spinal electromagnetic stimulation opens a window of synaptic plasticity in damaged spinal cord: role of NMDA receptors. J Neurophysiol..

[38] Müller-Dahlhaus F, Vlachos A. Unraveling the cellular and molecular mechanisms of repetitive magnetic stimulation. Front Mol Neurosci . 2013 ;6. 10.3389/fnmol.2013.00050 PMC3865432 24381540

[39] Opitz A, Windhoff M, Heidemann RM (2011). How the brain tissue shapes the electric field induced by transcranial magnetic stimulation. Neuroimage..

[40] Nakanishi T, Fujita Y, Tanaka T (2019). Anti-repulsive guidance molecule-a antibody treatment and repetitive transcranial magnetic stimulation have synergistic effects on motor recovery after spinal cord injury. Neurosci Lett..

[41] Niu T, Bennett CJ, Keller TL (2018). A Proof-of-Concept Study of Transcutaneous Magnetic Spinal Cord Stimulation for Neurogenic Bladder. Sci Rep..

[42] Leydeker M, Delva S, Tserlyuk I (2013). The effects of 15 Hz trans-spinal magnetic stimulation on locomotor control in mice with chronic contusive spinal cord injury. Electromagnetic Biology and Medicine..

[43] Li Z, Yao F, Cheng L (2019). Low frequency pulsed electromagnetic field promotes the recovery of neurological function after spinal cord injury in rats. J Orthop Res..

[44] Petrosyan HA, Alessi V, Hunanyan AS, et al. Spinal electro-magnetic stimulation combined with transgene delivery of neurotrophin NT-3 and exercise: novel combination therapy for spinal contusion injury. Journal of Neurophysiology. 2015;jn.00480.2015. 10.1152/jn.00480.2015 PMC4737407 26424579

[45] Guo F, Han X, Zhang J (2014). Repetitive transcranial magnetic stimulation promotes neural stem cell proliferation via the regulation of MiR-25 in a rat model of focal cerebral ischemia. PLoS ONE..

[46] Alizadeh A, Dyck SM, Karimi-Abdolrezaee S. Traumatic Spinal Cord Injury: An Overview of Pathophysiology, Models and Acute Injury Mechanisms. Front Neurol. 2019 ;10. 10.3389/fneur.2019.00282 PMC6439316 30967837

[47] Ahuja CS, Wilson JR, Nori S (2017). Traumatic spinal cord injury. Nature Reviews Disease Primers..

[48] Faulkner JR, Herrmann JE, Woo MJ (2004). Reactive Astrocytes Protect Tissue and Preserve Function after Spinal Cord Injury. J Neurosci..

[49] Bechler ME, Swire M, Ffrench-Constant C (2018). Intrinsic and adaptive myelination-A sequential mechanism for smart wiring in the brain. Dev Neurobiol..

[50] Hines JH, Ravanelli AM, Schwindt R (2015). Neuronal activity biases axon selection for myelination in vivo. Nat Neurosci..

[51] Cullen CL, Senesi M, Tang AD (2019). Low-intensity transcranial magnetic stimulation promotes the survival and maturation of newborn oligodendrocytes in the adult mouse brain. Glia..

[52] Filbin MT (2003). Myelin-associated inhibitors of axonal regeneration in the adult mammalian CNS. Nat Rev Neurosci..

[53] Pukos N, Goodus MT, Sahinkaya FR (2019). Myelin status and oligodendrocyte lineage cells over time after spinal cord injury: What do we know and what still needs to be unwrapped?. Glia..

[54] Tuszynski MH, Steward O (2012). Concepts and Methods for the Study of Axonal Regeneration in the CNS. Neuron..

[55] O’Shea TM, Burda JE, Sofroniew MV (2017). Cell biology of spinal cord injury and repair. J Clin Invest..

[56] Gaudet AD, Fonken LK (2018). Glial Cells Shape Pathology and Repair After Spinal Cord Injury. Neurotherapeutics..

[57] Abbasnia K, Ghanbari A, Abedian M (2015). The effects of repetitive transcranial magnetic stimulation on proliferation and differentiation of neural stem cells. Anatomy & Cell Biology..

[58] Dufor T, Grehl S, Tang AD (2019). Neural circuit repair by low-intensity magnetic stimulation requires cellular magnetoreceptors and specific stimulation patterns. Sci Adv.

[59] Cui M, Ge H, Zeng H (2019). Repetitive Transcranial Magnetic Stimulation Promotes Neural Stem Cell Proliferation and Differentiation after Intracerebral Hemorrhage in Mice. Cell Transplant..

[60] Zhang Z-C, Luan F, Xie C-Y (2015). Low-frequency transcranial magnetic stimulation is beneficial for enhancing synaptic plasticity in the aging brain. Neural Regen Res..

[61] Stein DM, Pineda JA, Roddy V (2015). Emergency Neurological Life Support: Traumatic Spine Injury. Neurocrit Care..

[62] Hachem LD, Ahuja CS, Fehlings MG (2017). Assessment and management of acute spinal cord injury: From point of injury to rehabilitation. The Journal of Spinal Cord Medicine..

[63] Seewoo BJ, Etherington SJ, Feindel KW (2018). Combined rTMS/fMRI Studies: An Overlooked Resource in Animal Models. Front Neurosci..

[64] Nardone R, Florea C, Höller Y (2017). Rodent, large animal and non-human primate models of spinal cord injury. Zoology (Jena)..

[65] Soderblom C, Luo X, Blumenthal E (2013). Perivascular Fibroblasts Form the Fibrotic Scar after Contusive Spinal Cord Injury. J Neurosci..

[66] Zhu Y, Soderblom C, Krishnan V (2015). Hematogenous macrophage depletion reduces the fibrotic scar and increases axonal growth after spinal cord injury. Neurobiol Dis..

[67] Duncan GJ, Plemel JR, Assinck P (2018). Locomotor recovery following contusive spinal cord injury does not require oligodendrocyte remyelination. Nat Commun.

