# Supplementary material for: The Regenerative Effect of Trans-spinal Magnetic Stimulation After Spinal Cord Injury: Mechanisms and Pathways Underlying the Effect
Source: Neurotherapeutics. 2020 Aug 27;17(4):2069–88. doi: 10.1007/s13311-020-00915-5 (PMC7851265; doi:10.1007/s13311-020-00915-5)
Supplement: Supplementary file 1 — (PDF 473 kb) [file 13311_2020_915_MOESM1_ESM.pdf]

Supplement Table I Number of mice used under all paradigms analyzed.

Fig.1	Mouse line	Experiments	n
SCI	C57BL/6	IHC (15 + 90 DPI)	5+4
SCI+STM	C57BL/6	IHC (15 + 90 DPI)	5+4
Fig.2	Mouse line	Experiments	n
SCI	C57BL/6	Locotronic	7
SCI+STM	C57BL/6	Locotronic	6
Fig.3	Mouse line	Experiments	n
SCI	C57BL/6	Proteomics	4
SCI+STM	C57BL/6	Proteomics	4
Fig.4	Mouse line	Experiments	n
SCI	C57BL/6	IHC (MBP)	6
SCI+STM	C57BL/6	IHC (MBP)	5
SCI	hFoxJ1/C57BL/6	IHC (NeuN)	4
SCI+STM	hFoxJ1/C57BL/6	IHC (NeuN)	4
SCI	C57BL/6	BDA	3
SCI+STM	C57BL/6	BDA	3
Fig.5	Mouse line	Experiments	n
SCI	C57BL/6	Neurospheres	4
SCI+STM	C57BL/6	Neurospheres	4
SCI	hFoxJ1/C57BL/6	IHC	5
SCI+STM	hFoxJ1/C57BL/6	IHC	5
Fig.6	Mouse line	Experiments	n
SCI	C57BL/6	Locotronic	9
SCI+STM	C57BL/6	Locotronic + IHC	9
SCI	C57BL/6	IHC	4
Fig.7	Mouse line	Experiments	n
SCI	C57BL/6	Locotronic	7
SCI+STM	C57BL/6	Locotronic + IHC	7
SCI	C57BL/6	IHC	5
Fig.8	Mouse line	Experiments	n
SCI	C57BL/6	Locotronic	7
SCI+STM	C57BL/6	Locotronic + IHC	7
SCI	C57BL/6	IHC	4
**Total**			**118**

Supplement Table II Statistical analyses performed in all Figures. DPI: Day Post Injury.

**Statistical analyses performed in all figures**			
**Figure and section**	**statistical test**	**comparison**	**p-value**
**1.D**	Two-tailed Mann-Whitney U test	Control/Stm<br>PDGFβ^{+} area 15d post SCI	0.0079
**1.G**	Two-tailed Mann-Whitney U test	Control/Stm<br>PDGFβ^{+}area 90d post SCI	0.0286
**1.J**	Two-tailed Mann-Whitney U test	Control/Stm<br>GFAP^{-}area 15d post SCI	0.0159
**1.M**	Two-tailed Mann-Whitney U test	Control/Stm<br>GFAP^{-}area 90d post SCI	0.0286
**1.P**	Two-tailed Mann-Whitney U test	Control/Stm<br>Iba1^{+} area15d post SCI	0.0079
**2.B**	Two-tailed Mann-Whitney U test	Control/Stm<br>Number of back legs errors<br>15d post SCI	0.0730
**2.C**	Two-tailed Mann-Whitney U test	Control/Stm<br>Total back legs errors time<br>15d post SCI	0.0530
**2.D**	Two-tailed Mann-Whitney U test	Control/Stm<br>Total crossing time<br>15d post SCI	0.0148
**2.E**	Two-tailed Mann-Whitney U test	Control/Stm<br>Number of back legs errors<br>30d post SCI	0.0006
**2.F**	Two-tailed Mann-Whitney U test	Control/Stm<br>Total back legs errors time<br>30d post SCI	0.0221
**2.G**	Two-tailed Mann-Whitney U test	Control/Stm<br>Total crossing time<br>30d post SCI	0.0047

**4.C**	Two-tailed Mann-Whitney U test	Control/Stm<br>MBP^{-}area 15d post SCI	0.0286
**4.F**	Two-tailed Mann-Whitney U test	Control/Stm<br>$NeuN^{+}$cells<br>15d post SCI	0.0286
**5.B**	Two-tailed Mann-Whitney U test	Control/Stm<br>Number of primary neurospheres<br>15d post SCI	0.0286
**5.C**	Two-tailed Mann-Whitney U test	Control/Stm<br>mean fluorescence intensity of tomato+ cells<br>15d post SCI	0.0286
**5.H**	Two-tailed Mann-Whitney U test	Control/Stm<br>Pourcentage of recombined Tomato+-GFAP+/Tomato+ cells<br>15d post SCI	0.0294
**5.K**	Two-tailed Mann-Whitney U test	Control/Stm<br>Pourcentage of recombined Tomato+-Sox10+/Tomato+ cells<br>15d post SCI	0.0286
**6.B**	Two-tailed Mann-Whitney U test	Chronical context stimulation<br>Control/Stm 25d post SCI<br>Number of back legs errors	0.0070
**6.B**	Two-tailed Mann-Whitney U test	Chronical context stimulation<br>Control/Stm 40d post SCI<br>Number of back legs errors	0.0076
**6.C**	Two-tailed Mann-Whitney U test	Chronical context stimulation<br>Control/Stm 25d post SCI<br>Total back legs errors time	0.0056
**6.C**	Two-tailed Mann-Whitney U test	Chronical context stimulation<br>Control/Stm 40d post SCI<br>Total back legs errors time	0.0120
**6.D**	Two-tailed Mann-Whitney U test	Chronical context stimulation<br>Control/Stm 25d post SCI<br>Total crossing time	0.0041

**6.D**	Two-tailed Mann-Whitney U test	Chronical context stimulation<br>Control/Stm 40d post SCI<br>Total crossing time	0.0016
**6.G**	Two-tailed Mann-Whitney U test	Chronical context stimulation<br>Control/Stm<br>PDGFβ^{+}area 90d post SCI	0.0286
**6.J**	Two-tailed Mann-Whitney U test	Chronical context stimulation<br>Control/Stm<br>GFAP^{-}area 15d post SCI	0.0079
**7.B**	Two-tailed Mann-Whitney U test	Chronical context stimulation<br>Control/Stm 25d post SCI<br>Number of back legs errors	0.0021
**7.B**	Two-tailed Mann-Whitney U test	Chronical context stimulation<br>Control/Stm 40d post SCI<br>Number of back legs errors	0.0021
**7.C**	Two-tailed Mann-Whitney U test	Chronical context stimulation<br>Control/Stm 25d post SCI<br>Total back legs errors time	0.0111
**7.C**	Two-tailed Mann-Whitney U test	Chronical context stimulation<br>Control/Stm 40d post SCI<br>Total back legs errors time	0.0111
**7.D**	Two-tailed Mann-Whitney U test	Chronical context stimulation<br>Control/Stm 25d post SCI<br>Total crossing time	0.0006
**7.D**	Two-tailed Mann-Whitney U test	Chronical context stimulation<br>Control/Stm 40d post SCI<br>Total crossing time	0.0026
**7.G**	Two-tailed Mann-Whitney U test	Chronical context stimulation<br>Control/Stm<br>PDGFβ^{+}area 90d post SCI	0.0043
**8.B**	Two-tailed Mann-Whitney U test	Chronical context stimulation<br>Control/Stm 25d post SCI<br>Number of back legs errors	0.0262

**8.B**	Two-tailed Mann-Whitney U test	Chronical context stimulation<br>Control/Stm 40d post SCI<br>Number of back legs errors	0.0101
**8.C**	Two-tailed Mann-Whitney U test	Chronical context stimulation<br>Control/Stm 25d post SCI<br>Total back legs errors time	0.8357
**8.C**	Two-tailed Mann-Whitney U test	Chronical context stimulation<br>Control/Stm 40d post SCI<br>Total back legs errors time	0.2468
**8.D**	Two-tailed Mann-Whitney U test	Chronical context stimulation<br>Control/Stm 25d post SCI<br>Total crossing time	0.2593
**8.D**	Two-tailed Mann-Whitney U test	Chronical context stimulation<br>Control/Stm 40d post SCI<br>Total crossing time	0.0732
**8.G**	Two-tailed Mann-Whitney U test	Chronical context stimulation<br>Control/Stm<br>PDGFβ^{+}area 90d post SCI	0.0286
**S1.A**	Two-tailed Mann-Whitney U test	Chronical context stimulation<br>Control 1d/Control 10d post SCI<br>Number of back legs errors	0.2086
**S1.B**	Two-tailed Mann-Whitney U test	Chronical context stimulation<br>Control 1d/Control 10d post SCI<br>Total back legs errors time	0.6529
**S1.C**	Two-tailed Mann-Whitney U test	Chronical context stimulation<br>Control 1d/Control 10d post SCI<br>Total crossing time	0.1590
**S1.D**	Two-tailed Mann-Whitney U test	Chronical context stimulation<br>Control 1d/Control 10d post SCI<br>Number of back legs errors	0.9490
**S1.E**	Two-tailed Mann-Whitney U test	Chronical context stimulation<br>Control 1d/Control 10d post SCI<br>Total back legs errors time	0.8979

**S1.F**	Two-tailed Mann-Whitney U test	Chronical context stimulation<br>Control 1d/Control 10d post SCI<br>Total crossing time	0.712
**S2.A**	Two-tailed Mann-Whitney U test	Control/Control<br>PDGFβ^{+} area 15d/90d post SCI	0.2857
**S2.A**	Two-tailed Mann-Whitney U test	Stm/Stm<br>PDGFβ^{+}area 15d/90d post SCI	0.063
**S2.B**	Two-tailed Mann-Whitney U test	Control/Control<br>GFAP^{-}area 15d/90d post SCI	1
**S2.B**	Two-tailed Mann-Whitney U test	Stm/Stm<br>GFAP^{-} area 15d/90d post SCI	0.11
**S3.A**	Two-tailed Mann-Whitney U test	Chronical context stimulation<br>Control 10d/Control 10d post SCI<br>Number of back legs errors	0.43
**S3.A**	Two-tailed Mann-Whitney U test	Chronical context stimulation<br>Stm 25d/Stm 25d post SCI<br>Number of back legs error	0.3933
**S3.A**	Two-tailed Mann-Whitney U test	Chronical context stimulation<br>Stm 40d/Stm 40d post SCI<br>Number of back legs error	0.7199
**S3.B**	Two-tailed Mann-Whitney U test	Chronical context stimulation<br>Control 10d/Control 10d post SCI<br>Total back legs errors time	0.0389
**S3.B**	Two-tailed Mann-Whitney U test	Chronical context stimulation<br>Stm 25d/Stm 25d post SCI<br>Total back legs errors time	0.0618
**S3.B**	Two-tailed Mann-Whitney U test	Chronical context stimulation<br>Stm 40d/Stm 40d post SCI<br>Total back legs errors time	0.83
**S3.C**	Two-tailed Mann-Whitney U test	Chronical context stimulation<br>Control 10d/Control 10d post SCI<br>Total crossing time	0.7577

**S3.C**	Two-tailed Mann-Whitney U test	Chronical context stimulation<br>Stm 25d/Stm 25d post SCI<br>Total crossing time	0.2429
**S3.C**	Two-tailed Mann-Whitney U test	Chronical context stimulation<br>Stm 40d/Stm 40d post SCI<br>Total crossing time	0.1326
**S3.D**	Two-tailed Mann-Whitney U test	Chronical context stimulation<br>Control 10d/Control 10d post SCI<br>Number of back legs errors	0.43
**S3.D**	Two-tailed Mann-Whitney U test	Chronical context stimulation<br>Stm 25d/Stm 25d post SCI<br>Number of back legs errors	0.0033
**S3.D**	Two-tailed Mann-Whitney U test	Chronical context stimulation<br>Stm 40d/Stm 40d post SCI<br>Number of back legs errors	0.0080
**S3.E**	Two-tailed Mann-Whitney U test	Chronical context stimulation<br>Control 10d/Control 10d post SCI<br>Total back legs errors time	0.4908
**S3.E**	Two-tailed Mann-Whitney U test	Chronical context stimulation<br>Stm 25d/Stm 25d post SCI<br>Total back legs errors time	0.011
**S3.E**	Two-tailed Mann-Whitney U test	Chronical context stimulation<br>Stm 40d/Stm 40d post SCI<br>Total back legs errors time	0.1199
**S3.F**	Two-tailed Mann-Whitney U test	Chronical context stimulation<br>Control 10d/Control 10d post SCI<br>Total crossing time	0.0311
**S3.F**	Two-tailed Mann-Whitney U test	Chronical context stimulation<br>Stm 25d/Stm 25d post SCI<br>Total crossing time	0.00198

**S3.F**	Two-tailed Mann-Whitney U test	Chronical context stimulation<br>Stm 40d/Stm 40d post SCI<br>Total crossing time	0.0078
**Table S3**	One-Way ANOVA	Protein quantification	Table S2 :<br>peptide features<br>p-value.q-value<0.05<br>power greater than 0.8<br>Were retained

Supplement Table III Table of proteins deregulated in Stm (rTSMS treated) mice 15 days after SCI from spinal cord samples.

This table lists the protein abbreviation and full name of the 156 proteins upregulated after rTSMS treatment 15 days after SCI. Quantifications are expressed as average ± SEM. N= 4 animals per group. Statistical evaluations were based on one-way ANOVA.

Accession	Description	Peptide count	Confidence score	Anova (p)	Max fold change	Highest mean condition	Lowest mean condition
1433B_MOUSE	14-3-3 protein beta/alpha	3	164,22	2,75E-03	1,14	Stm	Control
1433E_MOUSE	14-3-3 protein epsilon	4	179,93	4,17E-03	1,15	Stm	Control
1433G_MOUSE	14-3-3 protein gamma	3	172,47	1,19E-02	1,09	Stm	Control
1433Z_MOUSE	14-3-3 protein zeta/delta	2	118,54	1,94E-03	1,20	Stm	Control
2AAA_MOUSE	Serine/threonine-protein phosphatase 2A 65 kDa regulatory subunit A alpha isoform	2	85,07	5,27E-03	1,37	Stm	Control
AATC_MOUSE	Aspartate aminotransferase, cytoplasmic	7	233,63	8,06E-03	1,10	Stm	Control
AATM_MOUSE	Aspartate aminotransferase, mitochondrial	3	221,67	6,49E-04	1,15	Stm	Control
ACTB_MOUSE	Actin, cytoplasmic 1	6	241,07	1,26E-02	1,12	Stm	Control
ACTZ_MOUSE	Alpha-centractin	3	83,98	3,40E-03	1,39	Stm	Control
ADT1_MOUSE	ADP/ATP translocase 1	2	87,29	1,39E-03	1,26	Stm	Control
AINX_MOUSE	Alpha-internexin	9	532,89	2,66E-04	1,18	Stm	Control
ALBU_MOUSE	Serum albumin	2	82,65	9,05E-03	1,49	Stm	Control
ALDOA_MOUSE	Fructose-bisphosphate aldolase A	9	513,11	2,18E-03	1,13	Stm	Control
AT1A3_MOUSE	Sodium/potassium-transporting ATPase subunit alpha-3	2	64,68	1,34E-03	2,00	Stm	Control
AT1B1_MOUSE	Sodium/potassium-transporting ATPase subunit beta-1	4	188,62	4,36E-04	1,22	Stm	Control
AT1B2_MOUSE	Sodium/potassium-transporting ATPase subunit beta-2	3	147,33	5,93E-03	1,24	Stm	Control
AT5F1_MOUSE	ATP synthase F(0) complex subunit B1, mitochondrial	4	202,94	8,56E-04	1,21	Stm	Control
ATP5H_MOUSE	ATP synthase subunit d, mitochondrial	6	342,49	9,64E-05	1,31	Stm	Control
ATPA_MOUSE	ATP synthase subunit alpha, mitochondrial	8	327,31	1,71E-04	1,13	Stm	Control
ATPB_MOUSE	ATP synthase subunit beta, mitochondrial	3	213,71	8,55E-03	1,12	Stm	Control
ATPD_MOUSE	ATP synthase subunit delta, mitochondrial	2	115,07	3,70E-04	1,29	Stm	Control
ATPO_MOUSE	ATP synthase subunit O, mitochondrial	4	175,28	5,06E-04	1,33	Stm	Control
BACH_MOUSE	Cytosolic acyl coenzyme A thioester hydrolase	2	110,73	1,20E-02	1,35	Stm	Control
BASP1_MOUSE	Brain acid soluble protein 1	5	276,76	9,68E-03	1,38	Stm	Control
CALM1_MOUSE	Calmodulin-1	3	154,45	7,58E-05	1,44	Stm	Control
CALR_MOUSE	Calreticulin	2	76,09	3,86E-03	1,46	Stm	Control
CDC42_MOUSE	Cell division control protein 42 homolog	2	71,33	3,49E-03	1,30	Stm	Control
CEND_MOUSE	Cell cycle exit and neuronal differentiation protein 1	5	326,46	9,24E-04	1,21	Stm	Control
CH60_MOUSE	60 kDa heat shock protein, mitochondrial	3	189,48	6,79E-03	1,24	Stm	Control
CISY_MOUSE	Citrate synthase, mitochondrial	2	55,19	3,63E-03	1,14	Stm	Control
CN37_MOUSE	2',3'-cyclic-nucleotide 3'-phosphodiesterase	7	331,91	1,69E-03	1,17	Stm	Control
COF1_MOUSE	Cofilin-1	5	316,65	2,10E-04	1,50	Stm	Control
CSRP1_MOUSE	Cysteine and glycine-rich protein 1	6	267,53	2,62E-03	1,32	Stm	Control
DEST_MOUSE	Destrin	2	138,95	1,50E-03	1,28	Stm	Control
DHE3_MOUSE	Glutamate dehydrogenase 1, mitochondrial	4	167,98	2,67E-03	1,27	Stm	Control
DLDH_MOUSE	Dihydrolipoyl dehydrogenase, mitochondrial	4	222,37	1,34E-04	1,31	Stm	Control
DPYL2_MOUSE	Dihydropyrimidinase-related protein 2	5	296,38	8,72E-03	1,10	Stm	Control
EF1A2_MOUSE	Elongation factor 1-alpha 2	2	97,19	3,86E-03	1,20	Stm	Control
ENOA_MOUSE	Alpha-enolase	9	518,89	1,18E-03	1,24	Stm	Control
ENOG_MOUSE	Gamma-enolase	4	236,47	2,05E-03	1,16	Stm	Control
ES1_MOUSE	ES1 protein homolog, mitochondrial	2	81,52	2,44E-04	1,26	Stm	Control
ETFA_MOUSE	Electron transfer flavoprotein subunit alpha, mitochondrial	2	107,27	9,34E-03	1,19	Stm	Control
ETFB_MOUSE	Electron transfer flavoprotein subunit beta	2	78,14	2,03E-04	1,22	Stm	Control
FUMH_MOUSE	Fumarate hydratase, mitochondrial	2	65,21	1,32E-03	1,22	Stm	Control
G3P_MOUSE	Glyceraldehyde-3-phosphate dehydrogenase	2	95,58	1,91E-03	1,18	Stm	Control
G6PI_MOUSE	Glucose-6-phosphate isomerase	5	151,7	1,18E-03	1,18	Stm	Control
GABT_MOUSE	4-aminobutyrate aminotransferase, mitochondrial	3	123,78	1,16E-02	1,15	Stm	Control
GBB2_MOUSE	Guanine nucleotide-binding protein G(I)/G(S)/G(T) subunit beta-2	4	148,19	8,66E-03	1,26	Stm	Control

GDIB_MOUSE	Rab GDP dissociation inhibitor beta	2	85,22	4,33E-03	1,32	Stm	Control
GLNA_MOUSE	Glutamine synthetase	5	220,02	5,93E-04	1,19	Stm	Control
GLO2_MOUSE	Hydroxyacylglutathione hydrolase, mitochondrial	2	73,25	6,48E-03	1,24	Stm	Control
GLTP_MOUSE	Glycolipid transfer protein	3	140,24	1,26E-03	1,32	Stm	Control
GNAO_MOUSE	Guanine nucleotide-binding protein G(o) subunit alpha	4	176,2	2,92E-03	1,20	Stm	Control
GPDA_MOUSE	Glycerol-3-phosphate dehydrogenase [NAD(+)], cytoplasmic	3	133,73	1,04E-02	1,26	Stm	Control
GSTM1_MOUSE	Glutathione S-transferase Mu 1	6	227,21	5,84E-03	1,35	Stm	Control
GSTM5_MOUSE	Glutathione S-transferase Mu 5	4	161,65	5,03E-03	1,39	Stm	Control
H2B1B_MOUSE	Histone H2B type 1-B	3	77,17	1,08E-02	1,16	Stm	Control
H3C_MOUSE	Histone H3.3C	2	59,59	5,47E-03	1,20	Stm	Control
HSP7C_MOUSE	Heat shock cognate 71 kDa protein	3	112,03	6,29E-03	1,19	Stm	Control
K1H1_MOUSE	Keratin, type I cuticular Ha1	4	158,25	1,40E-02	16,27	Stm	Control
KAD1_MOUSE	Adenylate kinase isoenzyme 1	4	133,33	1,57E-03	1,27	Stm	Control
KAD3_MOUSE	GTP:AMP phosphotransferase AK3, mitochondrial	2	60,94	3,60E-03	1,12	Stm	Control
KCC2G_MOUSE	Calcium/calmodulin-dependent protein kinase type II subunit gamma	2	105,73	1,49E-02	1,89	Stm	Control
KCRB_MOUSE	Creatine kinase B-type	4	148,96	7,04E-03	1,14	Stm	Control
LDHB_MOUSE	L-lactate dehydrogenase B chain	6	304,38	1,50E-03	1,13	Stm	Control
LIS1_MOUSE	Platelet-activating factor acetylhydrolase IB subunit alpha	3	117,76	3,77E-03	1,23	Stm	Control
MBP_MOUSE	Myelin basic protein	5	282,58	2,76E-03	1,23	Stm	Control
MDHC_MOUSE	Malate dehydrogenase, cytoplasmic	2	127,62	2,59E-03	1,14	Stm	Control
MDHM_MOUSE	Malate dehydrogenase, mitochondrial	5	223,45	2,35E-04	1,15	Stm	Control
MIC19_MOUSE	MICOS complex subunit Mic19	2	92,91	1,56E-03	1,25	Stm	Control
MIC25_MOUSE	MICOS complex subunit Mic25	2	117,11	4,33E-03	1,45	Stm	Control
MK03_MOUSE	Mitogen-activated protein kinase 3	2	61,84	8,87E-03	1,17	Stm	Control
MOG_MOUSE	Myelin-oligodendrocyte glycoprotein	2	110,16	7,56E-04	1,10	Stm	Control
NDKB_MOUSE	Nucleoside diphosphate kinase B	2	60,38	2,35E-03	1,24	Stm	Control
NDUA8_MOUSE	NADH dehydrogenase [ubiquinone] 1 alpha subcomplex subunit 8	2	61,97	8,78E-03	1,26	Stm	Control
NDUA9_MOUSE	NADH dehydrogenase [ubiquinone] 1 alpha subcomplex subunit 9, mitochondrial	2	72,03	5,06E-03	1,36	Stm	Control
NDUAC_MOUSE	NADH dehydrogenase [ubiquinone] 1 alpha subcomplex subunit 12	3	156,13	1,03E-02	1,37	Stm	Control
NDUBA_MOUSE	NADH dehydrogenase [ubiquinone] 1 beta subcomplex subunit 10	3	127,41	3,24E-04	1,18	Stm	Control
NDUS2_MOUSE	NADH dehydrogenase [ubiquinone] iron-sulfur protein 2, mitochondrial	2	86,49	5,99E-04	1,15	Stm	Control
NDUS4_MOUSE	NADH dehydrogenase [ubiquinone] iron-sulfur protein 4, mitochondrial	2	45,01	6,99E-04	1,20	Stm	Control
NDUS6_MOUSE	NADH dehydrogenase [ubiquinone] iron-sulfur protein 6, mitochondrial	2	62,62	8,49E-04	2,09	Stm	Control
NDUS7_MOUSE	NADH dehydrogenase [ubiquinone] iron-sulfur protein 7, mitochondrial	2	54,93	8,82E-04	1,43	Stm	Control
NEUM_MOUSE	Neuromodulin	2	98,76	3,03E-03	1,20	Stm	Control
NFH_MOUSE	Neurofilament heavy polypeptide	2	97,9	4,38E-03	1,24	Stm	Control
NFM_MOUSE	Neurofilament medium polypeptide	5	256,4	5,88E-03	1,34	Stm	Control
ODPA_MOUSE	Pyruvate dehydrogenase E1 component subunit alpha, somatic form, mitochondrial	3	156,48	6,82E-03	1,10	Stm	Control
ODPB_MOUSE	Pyruvate dehydrogenase E1 component subunit beta, mitochondrial	2	92,53	2,58E-03	1,16	Stm	Control
ODPX_MOUSE	Pyruvate dehydrogenase protein X component, mitochondrial	2	63,45	4,25E-04	1,30	Stm	Control
OTUB1_MOUSE	Ubiquitin thioesterase OTUB1	2	118,79	4,29E-03	1,16	Stm	Control
PACN1_MOUSE	Protein kinase C and casein kinase substrate in neurons protein 1	3	189,39	6,54E-03	1,17	Stm	Control
PARK7_MOUSE	Protein/nucleic acid deglycase DJ-1	4	155,41	8,82E-04	1,30	Stm	Control
PCBP1_MOUSE	Poly(rC)-binding protein 1	2	44,01	3,50E-03	1,13	Stm	Control
PCSK1_MOUSE	ProSAAS	3	139,42	8,13E-03	1,20	Stm	Control
PEBP1_MOUSE	Phosphatidylethanolamine-binding protein 1	3	124,93	1,17E-03	1,21	Stm	Control
PGAM1_MOUSE	Phosphoglycerate mutase 1	4	219,97	5,70E-03	1,30	Stm	Control
PGK1_MOUSE	Phosphoglycerate kinase 1	2	156,09	1,38E-02	1,33	Stm	Control
PGM1_MOUSE	Phosphoglucomutase-1	3	121,78	6,29E-03	1,22	Stm	Control
PIMT_MOUSE	Protein-L-isoaspartate(D-aspartate) O-methyltransferase	3	150,1	8,52E-03	1,27	Stm	Control

PP1B_MOUSE	Serine/threonine-protein phosphatase PP1-beta catalytic subunit	2	46,76	1,41E-02	1,35	Stm	Control
PPIA_MOUSE	Peptidyl-prolyl cis-trans isomerase A	3	172,39	1,10E-03	1,24	Stm	Control
PRDX1_MOUSE	Peroxiredoxin-1	7	240,3	6,54E-04	1,16	Stm	Control
PRDX2_MOUSE	Peroxiredoxin-2	4	201,16	8,70E-05	1,26	Stm	Control
PRDX3_MOUSE	Thioredoxin-dependent peroxide reductase, mitochondrial	4	141,08	1,20E-04	1,39	Stm	Control
PRDX5_MOUSE	Peroxiredoxin-5, mitochondrial	4	196,71	2,74E-04	1,20	Stm	Control
PSB6_MOUSE	Proteasome subunit beta type-6	2	86,45	4,22E-04	1,29	Stm	Control
QCR1_MOUSE	Cytochrome b-c1 complex subunit 1, mitochondrial	4	173,31	4,60E-04	1,14	Stm	Control
QCR2_MOUSE	Cytochrome b-c1 complex subunit 2, mitochondrial	5	250,53	5,33E-03	1,19	Stm	Control
RAB10_MOUSE	Ras-related protein Rab-10	5	231,73	1,00E-03	1,29	Stm	Control
RAB1A_MOUSE	Ras-related protein Rab-1A	6	218,05	4,09E-05	1,28	Stm	Control
RAB2A_MOUSE	Ras-related protein Rab-2A	2	80,56	6,76E-03	1,19	Stm	Control
RAC1_MOUSE	Ras-related C3 botulinum toxin substrate 1	2	87,15	3,61E-04	1,33	Stm	Control
RAP1A_MOUSE	Ras-related protein Rap-1A	3	122,9	6,36E-04	1,33	Stm	Control
RAP1B_MOUSE	Ras-related protein Rap-1b	3	111,27	9,39E-03	1,33	Stm	Control
RHOB_MOUSE	Rho-related GTP-binding protein RhoB	3	128,67	1,45E-03	1,32	Stm	Control
RHOG_MOUSE	Rho-related GTP-binding protein RhoG	5	192,3	1,28E-03	1,29	Stm	Control
RL11_MOUSE	60S ribosomal protein L11	2	68,45	2,58E-03	1,42	Stm	Control
RLA0_MOUSE	60S acidic ribosomal protein P0	2	81,05	1,07E-03	1,30	Stm	Control
ROA2_MOUSE	Heterogeneous nuclear ribonucleoproteins A2/B1	2	61,53	1,14E-02	1,07	Stm	Control
SAHH2_MOUSE	S-adenosylhomocysteine hydrolase-like protein 1	3	93,64	3,77E-03	1,20	Stm	Control
SDHB_MOUSE	Succinate dehydrogenase [ubiquinone] iron-sulfur subunit, mitochondrial	2	93,16	9,25E-03	1,31	Stm	Control
SEPT7_MOUSE	Septin-7	2	69,56	2,63E-03	1,16	Stm	Control
SKP1_MOUSE	S-phase kinase-associated protein 1	3	127,35	5,68E-04	1,31	Stm	Control
SNAB_MOUSE	Beta-soluble NSF attachment protein	3	208,2	8,61E-04	1,23	Stm	Control
SODC_MOUSE	Superoxide dismutase [Cu-Zn]	5	282,47	5,45E-05	1,36	Stm	Control
SODM_MOUSE	Superoxide dismutase [Mn], mitochondrial	5	204,42	2,36E-04	1,36	Stm	Control
SSDH_MOUSE	Succinate-semialdehyde dehydrogenase, mitochondrial	2	71,83	6,21E-05	1,20	Stm	Control
STIP1_MOUSE	Stress-induced-phosphoprotein 1	2	61,75	5,99E-03	1,41	Stm	Control
STMN1_MOUSE	Stathmin	3	113,22	4,81E-03	1,18	Stm	Control
STXB1_MOUSE	Syntaxin-binding protein 1	4	174,82	2,03E-03	1,30	Stm	Control
SUCA_MOUSE	Succinate--CoA ligase [ADP/GDP-forming] subunit alpha, mitochondrial	2	56,35	4,57E-03	1,14	Stm	Control
SUCB1_MOUSE	Succinate--CoA ligase [ADP-forming] subunit beta, mitochondrial	4	239,08	1,74E-03	1,14	Stm	Control
SYUA_MOUSE	Alpha-synuclein	4	142,85	1,99E-03	1,45	Stm	Control
TAGL2_MOUSE	Transgelin-2	2	52,25	8,68E-03	1,35	Stm	Control
TAGL3_MOUSE	Transgelin-3	5	182,57	3,25E-03	1,29	Stm	Control
TAU_MOUSE	Microtubule-associated protein tau	2	81,84	3,29E-03	1,32	Stm	Control
THY1_MOUSE	Thy-1 membrane glycoprotein	3	189,3	4,08E-03	1,14	Stm	Control
TPIS_MOUSE	Triosephosphate isomerase	4	204,1	1,06E-03	1,21	Stm	Control
TPM3_MOUSE	Tropomyosin alpha-3 chain	2	88,86	3,46E-03	1,70	Stm	Control
TPPP3_MOUSE	Tubulin polymerization-promoting protein family member 3	2	43,04	2,55E-03	1,54	Stm	Control
UBE2N_MOUSE	Ubiquitin-conjugating enzyme E2 N	3	119,47	7,53E-04	1,20	Stm	Control
UCHL1_MOUSE	Ubiquitin carboxyl-terminal hydrolase isozyme L1	5	334,55	1,85E-03	1,24	Stm	Control
UCRI_MOUSE	Cytochrome b-c1 complex subunit Rieske, mitochondrial	4	130,12	4,32E-04	1,20	Stm	Control
VA0D1_MOUSE	V-type proton ATPase subunit d 1	2	66,33	3,53E-03	1,18	Stm	Control
VAMP2_MOUSE	Vesicle-associated membrane protein 2	2	105,07	1,25E-02	1,39	Stm	Control
VDAC1_MOUSE	Voltage-dependent anion-selective channel protein 1	7	445,09	2,79E-05	1,16	Stm	Control
VDAC2_MOUSE	Voltage-dependent anion-selective channel protein 2	4	248,68	1,33E-04	1,17	Stm	Control
VDAC3_MOUSE	Voltage-dependent anion-selective channel protein 3	3	154,51	8,10E-03	1,28	Stm	Control
VISL1_MOUSE	Visinin-like protein 1	2	100,5	1,76E-03	1,37	Stm	Control

ANXA2_MOUSE	Annexin A2	2	101,62	2,09E-03	1,44	Control	Stm
CADM4_MOUSE	Cell adhesion molecule 4	2	100,1	7,54E-03	1,26	Control	Stm
KCC2D_MOUSE	Calcium/calmodulin-dependent protein kinase type II subunit delta	2	86,17	1,37E-02	1,24	Control	Stm
MOES_MOUSE	Moesin	3	126,53	4,62E-03	1,52	Control	Stm
NCDN_MOUSE	Neurochondrin	6	233,42	9,45E-03	1,21	Control	Stm
NDUS1_MOUSE	NADH-ubiquinone oxidoreductase 75 kDa subunit, mitochondrial	2	89,86	8,62E-03	1,42	Control	Stm
SYN1_MOUSE	Synapsin-1	2	46,7	1,88E-03	1,36	Control	Stm
VIME_MOUSE	Vimentin	4	104,73	7,65E-03	1,29	Control	Stm

Supplement Fig I Locotronic test comparisons in chronical context one day after SCI and 10 days after SCI.

(A) Quantification of the number of back legs errors 1 day and 10 days after SCI in juvenile mice. (B) Quantification of the total back legs errors time 1 day and 10 days after SCI in juvenile mice. (C) Quantification of the total crossing time 1 day and 10 days after SCI in juvenile mice. N=7 animals. (D) Quantification of the number of back legs errors 1 day and 10 days after SCI in aged mice. (E) Quantification of the total back legs errors time 1 day and 10 days after SCI in aged mice. (F) Quantification of the total crossing time 1 day and 10 days after SCI in aged mice. N=7 animals. Quantifications are expressed as average $\pm$ SEM. ns= not significant.

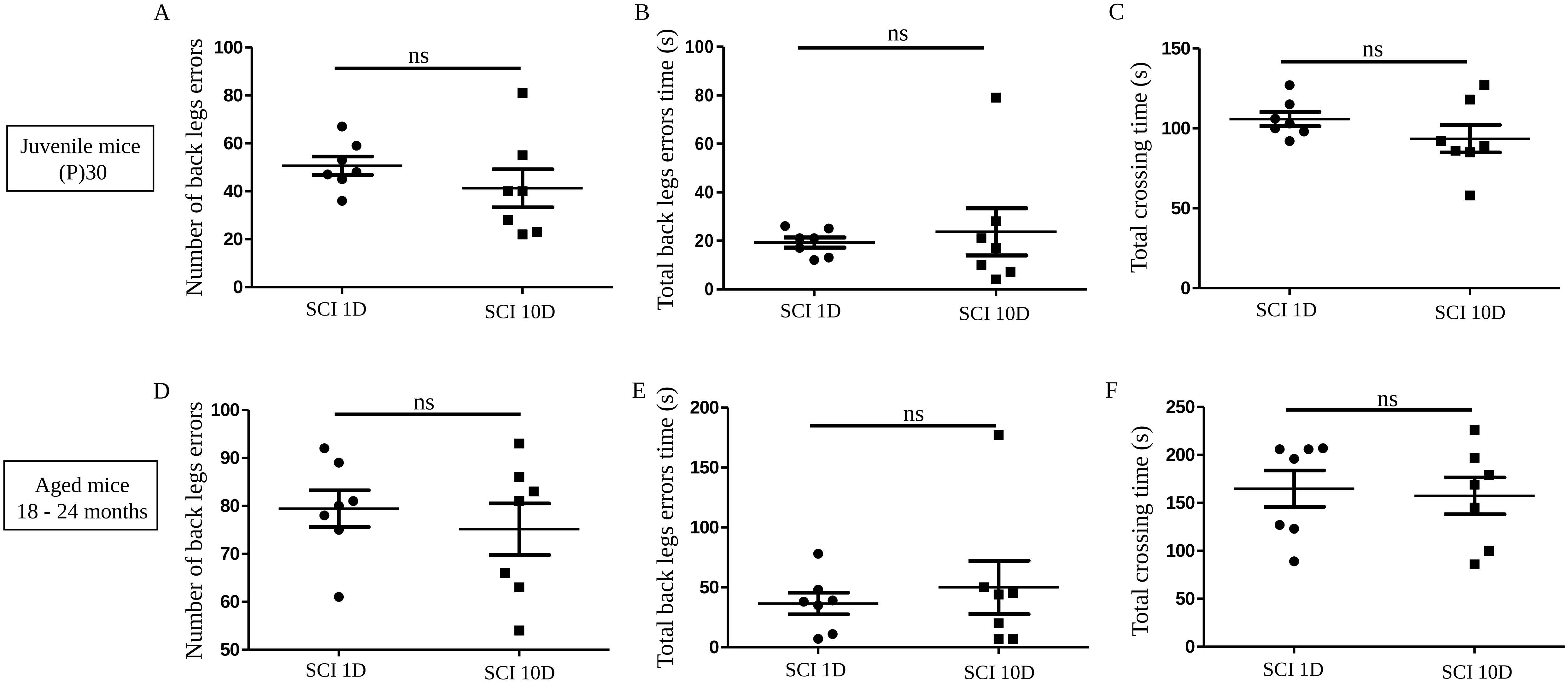
A
B
C
Juvenile mice
(P)30
Number of back legs errors
Total back legs errors time (s)
Total crossing time (s)
ns
SCI 1D
SCI 10D
D
E
F
Aged mice
18 - 24 months

Supplement Fig II Comparison of the fibrotic and glial components of the scar 15 days after SCI and 90 days after SCI.

(A) Quantification of PDGFRβ+ area 15 and 90 days after SCI in Control (SCI) mice and Stm (rTSMS treated) animals. (B) Quantification of GFAP negative area (GFAP-) 15 and 90 days after SCI in Control (SCI) mice and Stm (rTSMS treated) animals. N=5 animals per group at 15 days and N=4 animals per group at 90 days. Quantifications are expressed as average $\pm$ SEM. ns= not significant.

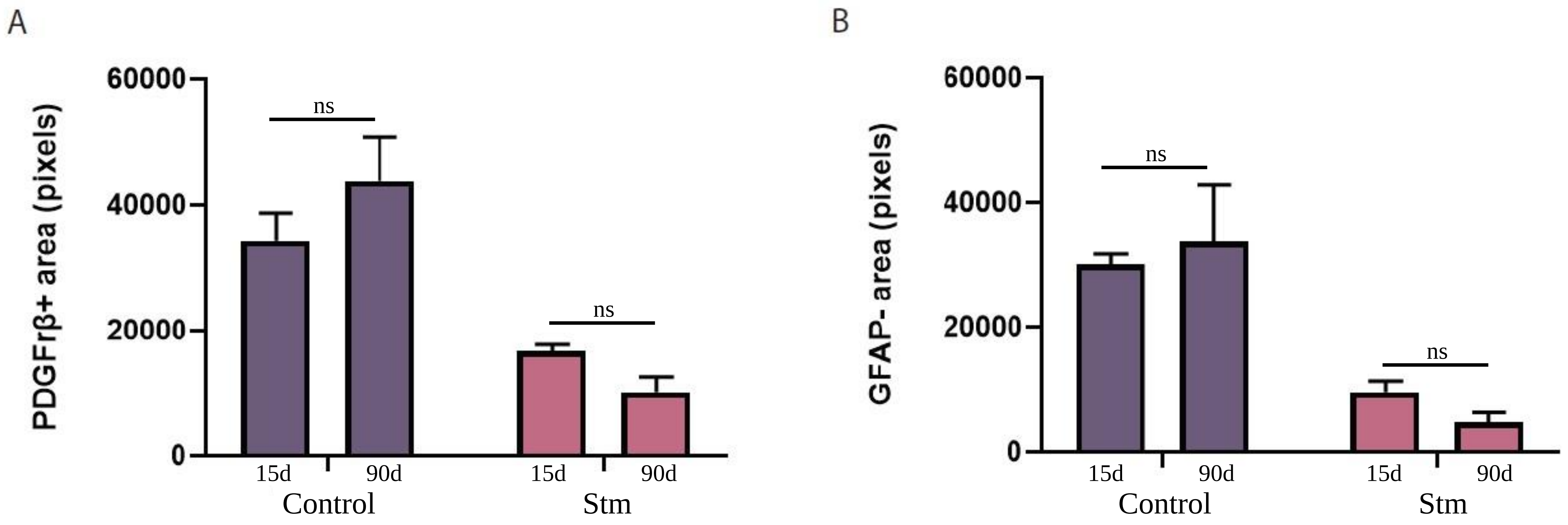
A
60000
40000
20000
0
PDGFrβ+ area (pixels)
ns
ns
15d
90d
15d
90d
Control
Stm
B
60000
40000
20000
0
GFAP- area (pixels)
ns
ns
15d
90d
15d
90d
Control
Stm

Supplement Fig III Comparison of the locotronic performances between adult and juvenile mice and between adult and aged mice in chronical context.

(A) Comparison of the number of back legs errors 10 days (Control SCI), 25 days and 40 days after SCI between adult and juvenile mice. (B) Comparison of the total back legs errors time 10 days, 25 days and 40 days after SCI between adult and juvenile mice. (C) Comparison of the total crossing time 10 days, 25 days and 40 days after SCI between adult and juvenile mice. N=9 animals at 10 days and 25 days and N=6 animals at 40 days for adult mice and N= 7 animals at 10 days and 25 days and N= 5 animals at 40 days for juvenile mice.

(D) Comparison of the number of back legs errors 10 days (Control SCI), 25 days and 40 days after SCI between adult and aged mice. (E) Comparison of the total back legs errors time 10 days, 25 days and 40 days after SCI between adult and aged mice. (F) Comparison of the total crossing time 10 days, 25 days and 40 days after SCI between adult and aged mice. N N=9 animals at 10 days and 25 days and N=6 animals at 40 days for adult mice and N= 7 animals at 10 days and 25 days and N= 5 animals at 40 days for aged mice. Quantifications are expressed as average $\pm$ SEM. ns=not significant * = $P < 0.05$; ** = $P < 0.01$.

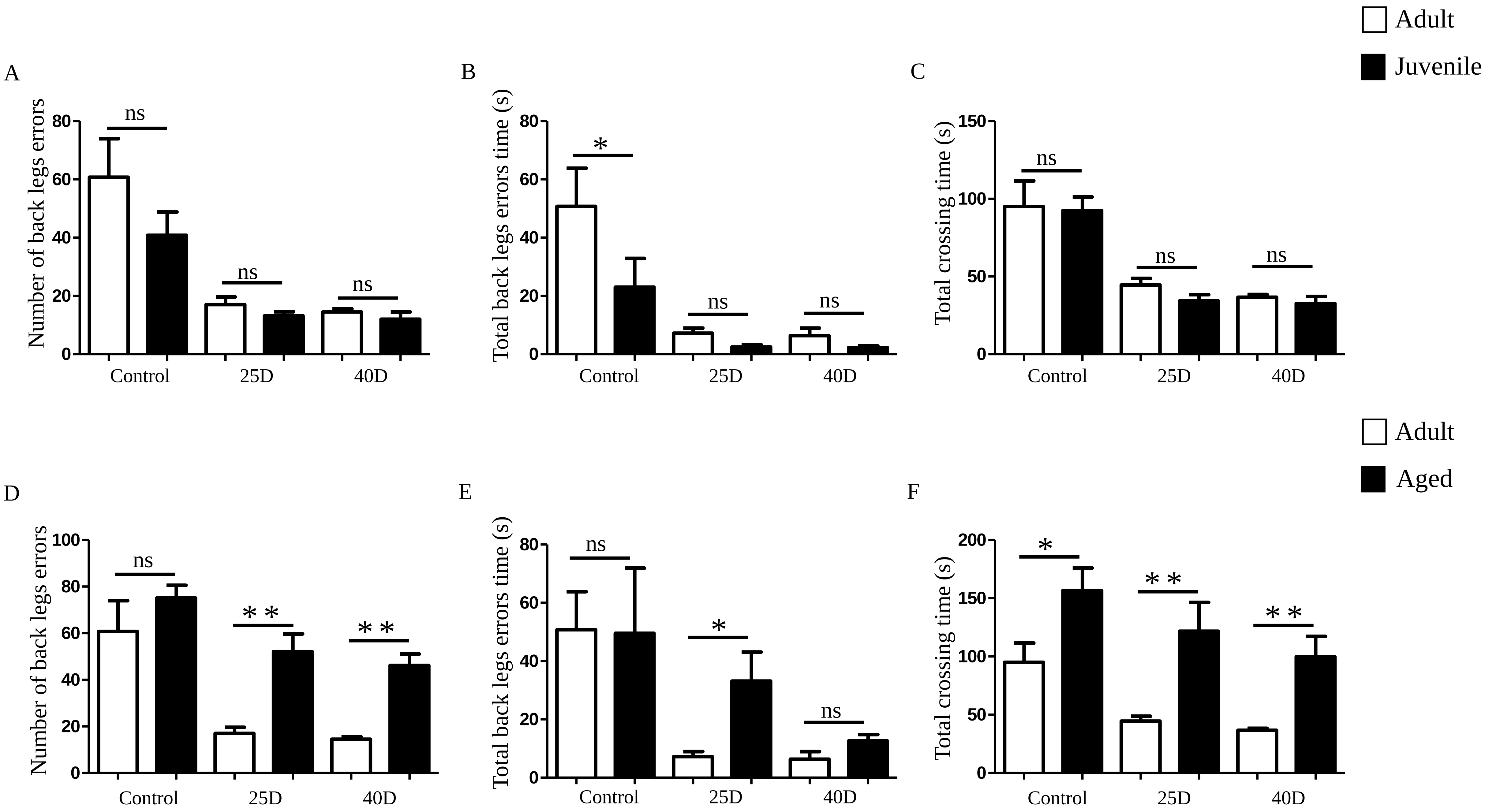
Adult
Juvenile
A
B
C
Number of back legs errors
Total back legs errors time (s)
Total crossing time (s)
ns
*
Control
25D
40D
Adult
Aged
D
E
F
* *

Movie I rTSMS based treatment enhances functional recovery, related to Fig. 2. **Videoclip of locotronic test 15 days after SCI in a Control mouse. This videoclip shows that SCI reduces locomotor function and induces spasticity.**

Movie II rTSMS based treatment enhances functional recovery, related to Fig. 2.

Videoclip of locotronic test 15 days after SCI in an rTSMS treated mouse. This videoclip shows that rTSMS treatment enhances locomotor function.
